# Construction of multilayered gene circuits using de-novo-designed synthetic transcriptional regulators in cell-free systems

**DOI:** 10.1186/s13036-024-00459-8

**Published:** 2024-11-05

**Authors:** Mingming Zhao, Jeongwon Kim, Jiayan Jiao, Yelin Lim, Xianai Shi, Shaobin Guo, Jongmin Kim

**Affiliations:** 1 College of Biological Science and Engineering, Fuzhou University, Fuzhou, Fujian 350108 China; 2 Department of Life Sciences, Pohang University of Science and Technology, Pohang, 37673 Korea; 3 Fujian Key Laboratory of Medical Instrument and Pharmaceutical Technology, Fuzhou University, Fuzhou, Fujian 350108 China; 4 International Joint Laboratory of Intelligent Health Care, Fuzhou University, Fuzhou, Fujian 350108 China

**Keywords:** Synthetic riboregulator, Transcriptional regulation, Computational design, Multilayered cascades, Synthetic logic circuits

## Abstract

**Background:**

De-novo-designed synthetic transcriptional regulators have great potential as the genetic parts for constructing complex multilayered gene circuits. The design flexibility afforded by advanced nucleic acid sequence design tools vastly expands the repertoire of regulatory elements for circuit design. In principle, the design space of synthetic regulators should allow for the construction of regulatory circuits of arbitrary complexity; still, the orthogonality and robustness of such components have not been fully elucidated, thereby limiting the depth and width of synthetic circuits.

**Results:**

In this work, we systematically explored the design strategy of synthetic transcriptional regulators, termed switchable transcription terminators. Specifically, by redesigning key sequence domains, we created a high-performance switchable transcription terminator with a maximum fold change of 283.11 upon activation by its cognate input RNA. Further, an automated design algorithm was developed for these elements to improve orthogonality for a complex multi-layered circuit construction. The resulting orthogonal switchable transcription terminators could be used to construct a three-layer cascade circuit and a two-input three-layer OR gate.

**Conclusions:**

We demonstrated a practical strategy for designing standardized regulatory elements and assembling modular gene circuits, ultimately laying the foundation for the streamlined construction of complex synthetic gene circuits.

**Supplementary Information:**

The online version contains supplementary material available at 10.1186/s13036-024-00459-8.

## Background

Synthetic biology, as a fast-developing field, offers new possibilities for biological [1–3] and medical applications [4, 5] by utilizing the principles of biological circuit design. Over the past two decades, significant progress has been made in designing and constructing synthetic circuits, including oscillators, bistable switches, and logic gates [6–9], which play crucial roles in cellular [10, 11] and cell-free regulatory networks [12–15]. While traditional protein-based regulators have been widely used in the circuit construction to fulfill the need of gene regulation, their limitations, such as resource consumption, unpredictability of cross-reactions, and cell toxicity, have spurred the exploration of nucleic acid-based gene regulators [16, 17]. 

In recent years, nucleic acid-based regulators have become a versatile tool for constructing synthetic biological systems due to their design flexibility, seamless integration into complex systems, and advantages over protein-based regulators, such as reduced resource usage, faster response rates, and faithful signal propagation [18, 19]. In particular, RNA-based regulators, such as riboswitches, riboregulators, and small RNAs, with their short half-life, minimal amino acid usage, compact encoding space, and the aid of growing insights into RNA biology [20, 21] and computational tools [22, 23], have the potential to greatly accelerate progress in synthetic biology. Driven by significant advancements in nucleic acid sequence design tools [24, 25], synthetic biology field has witnessed a remarkable expansion in the synthetic RNA-based regulatory elements, opening up new avenues for the construction of regulatory circuits with increased complexity.

Previously, Chappell et al. designed a small transcription activating RNA (STAR) based on bacterial transcription terminators [26]. They combined STARs with CRISPRi to construct logic gates for transcriptional regulation, creating novel RNA-based genetic circuits. Hong et al. then drew inspiration from STARs and designed switchable transcription terminators (SWTs) using a combination of engineered toehold sequence modifications and natural and synthetic terminators [27]. STAR and SWT share the design principles in that both STAR and SWT utilize strand displacement of rho-independent terminator in response to input RNA strand to allow transcription. STAR, as a landmark synthetic transcription regulator, introduced a large orthogonal library through the computational design of synthetic toehold domains [28]. SWT aimed to further expand the design space by focusing on the terminator domain including the mutant phage terminator T500 and other synthetic terminator variants [29]. 

Both STARs and SWTs exhibit a remarkable advantage of low leakage, which ensures a precise control over the transcriptional process. Despite this advantageous feature, constructing a library of orthogonal elements in synthetic RNA transcription regulators including STAR and SWT remains a challenge. These synthetic transcription regulators typically employ long input RNA sequences to increase the binding rates between the input and regulator element since the transcription termination is an irreversible step if it occurs before the arrival of input RNA, which in turn could increase the potential for crosstalk between different inputs and regulatory elements. Addressing this challenge is crucial to enhance the reliability and specificity of transcriptional control mediated by synthetic RNA transcription regulators. By unraveling the underlying mechanisms and developing innovative strategies to mitigate crosstalk, researchers can harness the full potential of these regulatory elements for more precise and predictable control of gene expression for a wide range of applications in synthetic biology and biotechnology.

Here, building on the design and application of SWTs and STARs, we systematically explored the design elements of SWTs, developed new algorithms for orthogonal library, and constructed regulatory circuits. The importance of different domains of SWTs was characterized and used to propose a design strategy for high-performance SWTs. Unlike some earlier works where the transcription terminator sequence was fixed, a number of variants on the terminator sequences were tested to elucidate the features of terminator sequences and to expand the designable space for sequences for further exploration. Moreover, in order to improve the orthogonality between SWTs, an automated design algorithm was developed to perform orthogonality test on different SWTs and trigger RNAs analogous to earlier work by Chappell et al. [28] With these orthogonal SWTs and trigger RNAs, the construction of a three-layer cascade circuit was demonstrated using only RNAs as inputs. In addition, a two-input three-layer OR gate was constructed using these SWTs, further demonstrating the design flexibility and application in synthetic logic circuits. We believe these results can provide valuable insights for integrating regulatory RNAs in the construction of multiplexed biological circuits with improved orthogonality, complexity, and bandwidth.

## Methods

### SWT design and NUPACK analysis

De-novo-designed synthetic transcription terminators, SWTs, were designed to provide the toehold region for trigger binding and the terminator region (typically T500 and its variants) followed by the 3-Way Junction dimeric Broccoli (3WJdB) for transcription outputs. NUPACK sequence design package [22] was used to design sequences for SWT, where the secondary structure of the toehold domain and the terminator stem domain of SWT were included for sequence assignment using the RNA parameters at 37 °C setting [30, 31]. The resulting SWT designs were evaluated for potential crosstalk with NUPACK with a maximum complex size of 2 and at a concentration of 10 nM each [32]. 

### Plasmid construction

All sequences and plasmids used in this study are listed in Supplementary Tables S1, S2, and S3. All DNA oligonucleotides were purchased from Fuzhou Sunya Biotechnology. The plasmid pSG-backbone contains the candidate SWT and 3WJdB driven by the T7 promoter based on the design of the pSG81. pSG81 contains a carbenicillin resistance gene and an origin of replication ColE1 to allow its passage through *E. coli*. All the gene fragments (Fuzhou Sunya Biotechnology) were ligated using Golden Gate assembly (#M0551S, New England Biolabs) and the ligated product was transformed into *E. coli* DH5α strain and cultured on LB agar plate with appropriate antibiotics (100 µg/mL carbenicillin, Shanghai yuanye Bio-Technology). Single colonies were inoculated in LB liquid medium with appropriate antibiotics, and cultured for 14 h at 37 °C, 220 rpm. A volume of 1 mL of cultured bacteria was taken for sequencing (Fuzhou Sunya Biotechnology). The bacterial strains with correct sequencing results were preserved, and the plasmids were extracted.

### In vitro transcription reaction

To prepare linearized templates for in vitro transcription, the plasmids were used as templates for polymerase chain reaction with primers to amplify regions including T7 promoter and desired transcript (#DC301-01, Vazyme Biotech). In vitro transcription reactions were prepared on ice with 5–40 nM of linearized DNA template, 40 µM of DFHBI-1T (#SML2697, Sigma-Aldrich), 0.5 mM of NTPs, 1.5 µL of T7 RNAP (50 U/µL), and 0.75 µL of ribonuclease inhibitor (#R301-03, Vazyme Biotech) in the reaction buffer (40 mM Tris-HCl pH 7.9, 6 mM MgCl_2_, 2 mM spermidine, 1 mM DTT) (#B9012, New England Biolabs) at a final volume of 30 µL.

### Fluorescence measurement and analysis

The in vitro transcription reaction was conducted in a 384-well plate (Agilent) with three replicates per experimental condition, including a control reaction where the DNA template was excluded. The reaction temperature was controlled at 37 °C and the fluorescence signal to determine the transcription level of 3WJdB was measured using a plate reader (Molecular Devices). The 3WJdB fluorescence (excitation/emission 472/507 nm) was taken 2 h after the start of the in vitro transcription reaction.

To normalize the signals from each batch, the fluorescence of the control reaction (referred to as background) was subtracted from the fluorescence of the experimental sets, termed normalized fluorescence (Eq. 1).

1 $$\begin{gathered} Normalized\,Fluorescence \hfill \\= Fluorescence-Background \hfill \\ \end{gathered}$$

The fold change for each SWT construct was defined as the normalized fluorescence in the presence of the cognate trigger (ON state) divided by the normalized fluorescence in the absence of the cognate trigger (OFF state) (Eq. 2).

2 $$\begin{gathered} Fold\,Change = \hfill \\ Normalized\,Fluorescenc{e_{ON}}/Normalized\,Fluorescenc{e_{OFF}} \hfill \\ \end{gathered}$$

A Welch’s *t*-test was applied to determine the statistical significance (*P* < 0.05 or 0.01) of the results obtained under different conditions.

### Algorithm for orthogonal SWT library

The algorithm for sequence generation of orthogonal SWTs was constructed based on NUPACK Python module (NUPACK 4.0) [22], and utilized libraries for multi-tube design [33]. The parameters used for free energy calculations are consistent with the NUPACK web application used for preliminary SWT characterization, and both reference the RNA energy parameters proposed by Mathews et al. [31] For each SWT, the stem-loop region was preserved as the T500 terminator sequence, and the toehold region was set to a random sequence of 40 bases. The GC-content of the toehold region was set to 50–60%. The trigger RNA was designed to be complementary to the toehold region and stem sequence of the corresponding SWT. The trigger RNA structure was defined to be linear, and the completely hybridized structure between the SWT and its corresponding trigger RNA was defined as the target complex structure. Three categories of test tubes were declared each containing (1) individual constructs, (2) the entire SWTs or trigger RNAs, and (3) pairs of SWT and trigger RNA. The concentration of each construct and their target complex followed the conditions of the in vitro experiments. For more detailed description of the algorithm section, please refer to the supplementary material.

## Results

### Design of SWTs

**Fig. 1:**
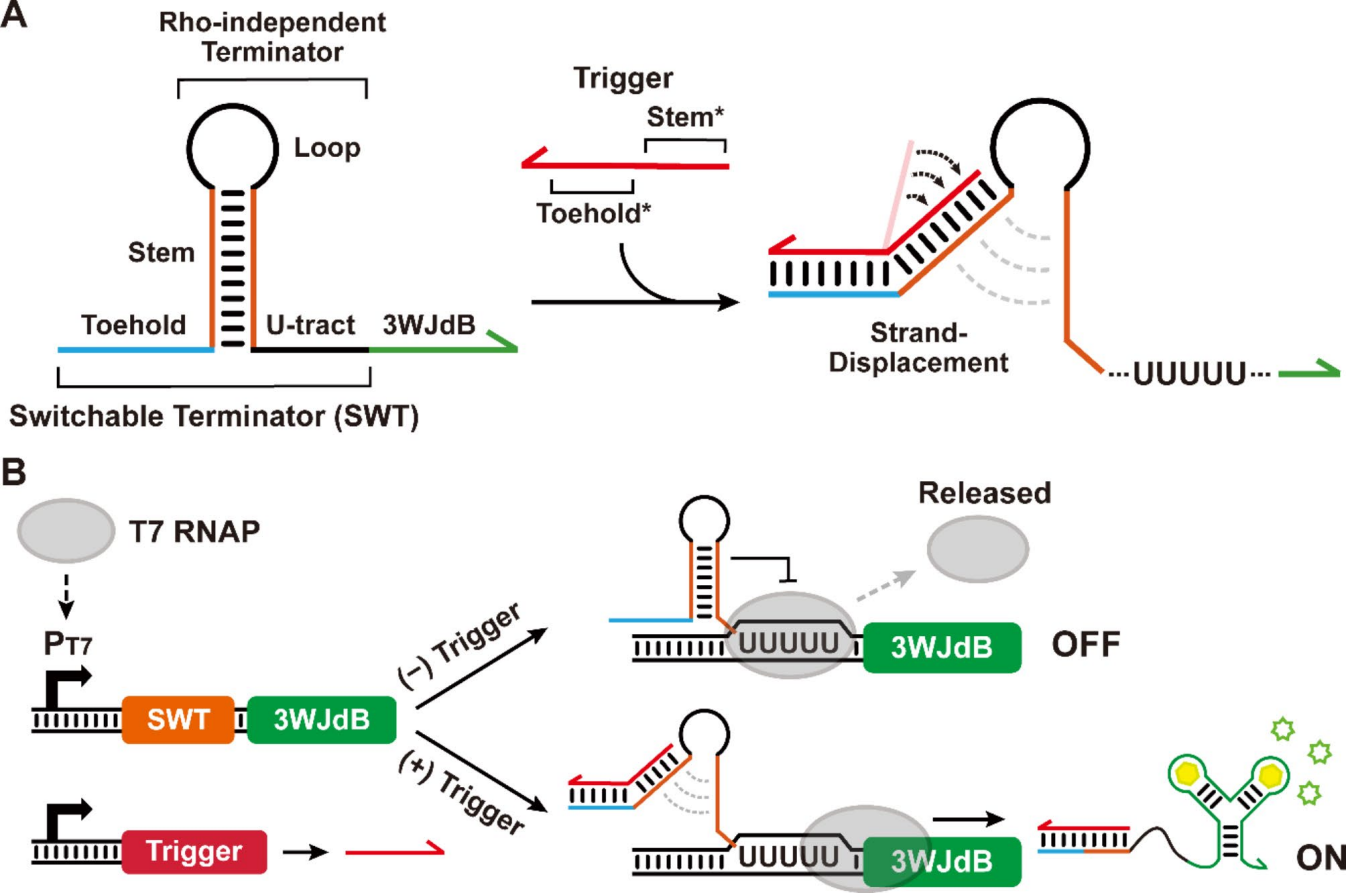
The design and mechanism of SWT. (**A**) A SWT consists of two main domains: a linear toehold region and a rho-independent terminator, which in turn encompasses a hairpin stem with a small loop, and a poly U-tract. Fluorescent aptamer 3WJdB is used as the reporter to characterize the transcription efficiency through the change of fluorescence output. A trigger RNA consists of sequences complementary to the stem and toehold regions (denoted by asterisks (*)). The hybridization of trigger RNA and SWT can disrupt the stable hairpin structure of rho-independent terminator to allow transcription of downstream gene. (**B**) In the absence of the trigger RNA, the terminator structure in SWT halts the transcription process (OFF). In the presence of the trigger RNA, the trigger RNA can initiate hybridization through the toehold region of SWT, and continue the branch migration to disrupt the formation of terminator stem, thereby allowing for the transcription of the downstream reporter (ON)

Previously, de-novo-designed synthetic transcription regulators, SWTs, were reported starting from several natural and synthetic terminators [27]. Here, we aim to further generalize the design principle of SWT by expanding the designable space of SWTs, thereby constructing a library of high-performance SWTs. First, we explore the key domains of SWT: a toehold region followed by a strong rho-independent transcription terminator that consists of a strong hairpin stem with a small loop region followed by the long poly-U tract [34] (Fig. 1A). In the absence of a cognate trigger RNA, the transcription terminator part of SWT forms a strong hairpin structure followed by poly-U tract, which terminates transcription by RNA polymerase (OFF state). On the other hand, when the trigger RNA is present, it can initiate hybridization with SWT at the toehold domain and continue branch migration to disrupt the formation of terminator hairpin, allowing RNA polymerase to continue transcription (ON state) (Fig. 1B and Supplementary Figure S1). To measure the transcriptional output from SWT, we used a fluorescent RNA aptamer, the 3WJdB [35], as the reporter, allowing for real-time fluorescence measurement of transcription activity.

### Design of trigger RNAs

Next, we aimed to investigate the trigger RNA designs with respect to the performance of regulating SWT. While SWT itself contains a very stable secondary structure in the form of transcription terminator stem, the trigger RNA needs to effectively interact with the toehold domain and the stem domain of SWT such that it can break the terminator structure formation to rescue transcription. To elucidate the trigger RNA design strategy, we screened 4 different trigger RNAs composed of a different combination of important domains to interact with SWT (Supplementary Figure S2A). Not surprisingly, trigger RNAs containing only the toehold region or the stem region were insufficient to disrupt the terminator of SWT and hence remain transcriptionally inactive, while those trigger RNA designs that encompass both the toehold and stem regions can efficiently turn on the SWT (Supplementary Figure S2B). The longest trigger RNA that also binds to the loop region showed a lower activation fold compared to the trigger RNA that does not have domains corresponding to the loop region, indicating that the elongated loop-binding domain may slightly interfere with the proper interaction of SWT and trigger RNAs. Based on these results, we chose to investigate further the trigger RNA designs that encompass just the toehold and stem regions of SWT.

### Optimization of SWT and the expansion of its designable space

To expand the designable space of SWTs, a set of different SWT variants was first constructed and analyzed. Even for SWTs with identical terminator sequences, the performances varied depending on the sequences in the toehold domains (Supplementary Figure S3). Since a strong initial interaction of SWT and trigger RNA could be an important factor that affect the SWT performance, we designed and tested several SWT variants consisting of toehold domains with a wide range of GC contents to investigate their impact.

**Fig. 2:**
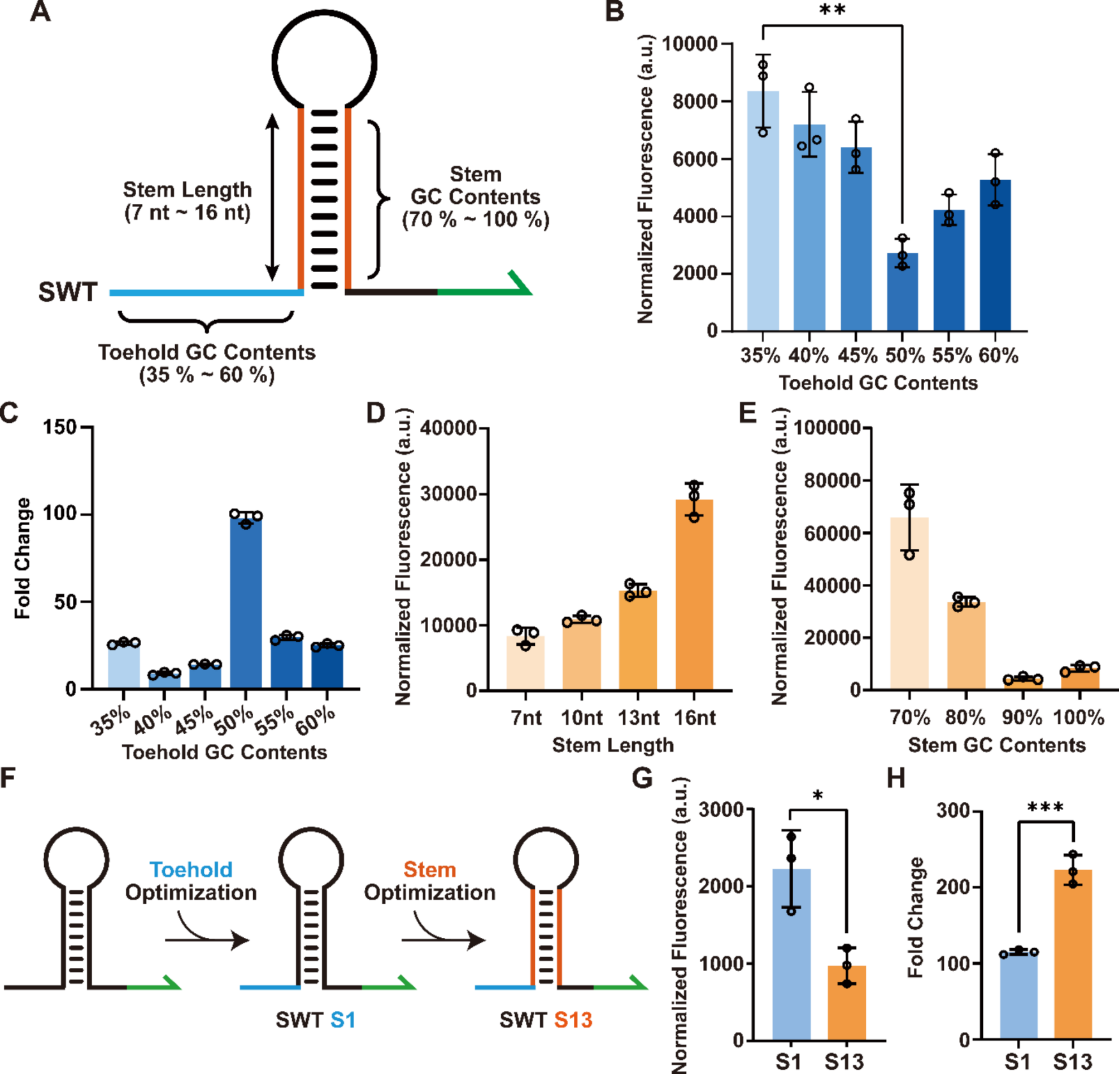
Characterization of SWTs with different design constraints. (**A**) Schematic of SWT with different toehold and stem sequence design constraints. (**B**) Leakage of SWTs (normalized fluorescence of OFF state) with different toehold region. The SWTs composed of different toehold region were designed with 5% GC decrement, and the differences of leakage values between them were compared. (**C**) Characterization of SWTs with different toehold GC contents. Fold change was measured with templates for SWTs at 10 nM and templates for trigger RNAs at 20 nM. (**D**, **E**) Fluorescence characterization was performed on SWTs with different stem regions. The leaky expression levels were measured with templates for SWTs at 5 nM. (**F**) Schematic of S1 and S13, combining the optimized toehold sequence with the redesigned stem sequence. (**G**, **H**) Fluorescence characterization was performed on S1 and S13. The data in G and H are the comparison of the leakage value (**G**) and ON/OFF ratio (**H**), respectively. All data shown are *n* = 3 independent biological replicates. For data in B, G, and H, Welch’s *t*-tests were performed on each construct, *P < 0.05, **P < 0.01, and ***P < 0.001. Error bars represent the standard deviation (s.d.) of three biological replicates

Specifically, a set of SWTs with toehold regions that have GC contents ranging from 35 to 60% were designed using NUPACK and tested in vitro (Fig. 2A). Also with NUPACK, we ensured that no secondary structure was formed in the toehold region to fulfill our design requirement. To identify the impact of GC contents within the toehold region on SWT performance, the fluorescence leakage levels in the OFF states and the maximum fold changes for different SWT concentrations were measured (Fig. 2B and C, Supplementary Figures S4, S5). The toehold region with the GC content at 50% exhibited the lowest level of leaky signal in the OFF state; however, the leaky signals increased as the GC contents decreased below 50% with the SWT with 35% GC contents showing highest leaky signal. The SWT with a maximum ON/OFF ratio of 98.06-fold was found to have 50% GC in its toehold region (Supplementary Figure S6), and therefore, we chose to further explore SWT designs with the same sequence composition in their toehold domains.

Previous work demonstrated that the stem-loop region in the terminator structure of SWT can be replaced with other terminator sequences while maintaining a large dynamic range and a low leakage level [27]. To characterize the impact of the length of the terminator stem region on leaky expression from SWTs, the SWT with the highest leakage (S6-GC: 35%) was chosen for further design modifications. The initial design used the stem sequence of T500 terminator, which consists of a strong 7-bp stem with 100% GC bases. We designed a series of stem sequences with 3-bp increments while maintaining all GC bases (Fig. 2A). Unexpectedly, increasing the stem length beyond 10-bp apparently increased the leaky expression from SWT possibly due to incomplete formation of stem or spurious binding of the stem with other regions of SWT (Fig. 2D). Still, a 10-bp terminator stem maintained a similar level of leaky expression as compared to the original T500 terminator stem. Starting from the new 10-bp terminator stem, more sequence designs could be explored, where the GC content in the stem region was varied from 70 to 100%. As expected, GC percentage lower than 90% led to increased leakage due to weakened stem stability for the terminator structure, while a 90% GC base composition could maintain a similar level of leakage as compared to all GC base pairs (Fig. 2E).

Through the design exploration of terminator stem regions, the stem regions were extended from 7-bp to 10-bp with less GC content requirement from 100 to 90%, while maintaining a similar leakage level. This amounts to increasing the sequence choice for stem regions by 80-fold. To verify these design choices, the optimal toehold sequence composition was combined with the terminator stem region with a GC content of 90% and a length of 10-bp to construct a new SWT, S13 (Fig. 2F). The SWT S13, with a stem sequence distinct from natural terminators, showed lower leakage and improved ON/OFF ratio compared to S1 (Fig. 2G and H, Supplementary Figure S7). Together, these design exploration results provide a valuable reference for the design of high-performance SWTs, expanding the designable space for a high-performance SWT library.

### Algorithmic design of orthogonal SWTs and construction of multilayered cascade circuits

The inherent programmability of RNA-based regulators provides the possibility of constructing large orthogonal libraries [36]. Despite its potential, the previous study only demonstrated a three-layer cascade reaction utilizing the STAR [28] and multilayered circuit construction with synthetic RNA regulators still remains a challenge [37]. The design flexibility of SWT, where, in principle, the toehold region can be arbitrarily chosen and the terminator stem region can be adjusted within bounds, can potentially solve the limitation of synthetic transcription regulators and further expand the range of realizable multilayered gene circuits.

To obtain orthogonal SWTs for multilayered circuit design, a set of linear toehold domain sequences consisting of 40-bases of random sequence were generated and screened for unwanted interactions with the terminator sequence domain. The candidate SWT sequences predicted to have toehold domains completely unpaired were further analyzed to find a new SWT. The resulting S14 and its trigger (T14) were analyzed in silico for orthogonality with respect to an existing SWT, S1. Simulation results predicted that T1, the trigger for S1, has a fairly strong binding to S14 (Supplementary Figures S8, S9). In vitro characterization also confirmed that the orthogonality between S1 and S14 was not sufficient to build a multilayered circuit (Supplementary Figure S10). Recognizing that an automated design algorithm could assist in the design process for an increased reliability of orthogonal SWT library, we developed a systematic SWT design algorithm using the Python module provided by NUPACK. NUPACK algorithms are formulated in terms of nucleic acid secondary structures and can evaluate equilibrium properties of a complex of nucleic acid strands. And therefore, NUPACK algorithm combined with in-house software pipelines can provide a valuable tool to design a library of orthogonal SWTs. After some iterations of different design choices and evaluating the candidates in silico, we employed the multi-tube design to ensure orthogonality while incorporating critical design features such as GC content. The multi-tube design is a component of the NUPACK design ensemble used for sequence design. In a single-tube design, sequences are simulated within a single in silico test tube, accounting for both the desired on-target complex and unintended off-target complexes. Based on this, the multi-tube design considers multiple test tubes with different combinations of switch and trigger sequences. This allows for the design of sequences that are expected to exhibit higher orthogonality across different pairs by setting the cognate switch-trigger combination as on-target, and non-cognate combinations as off-targets [33]. We also ensured that the complete trigger sequences were comprised of both the toehold and terminator domains, integral for the function of our synthetic transcriptional regulators. In silico screening and evaluation of the candidate SWTs from the design algorithm resulted in two novel SWTs, termed S15 and S16, which met the design requirements with little crosstalk (Supplementary Figure S11).

**Fig. 3:**
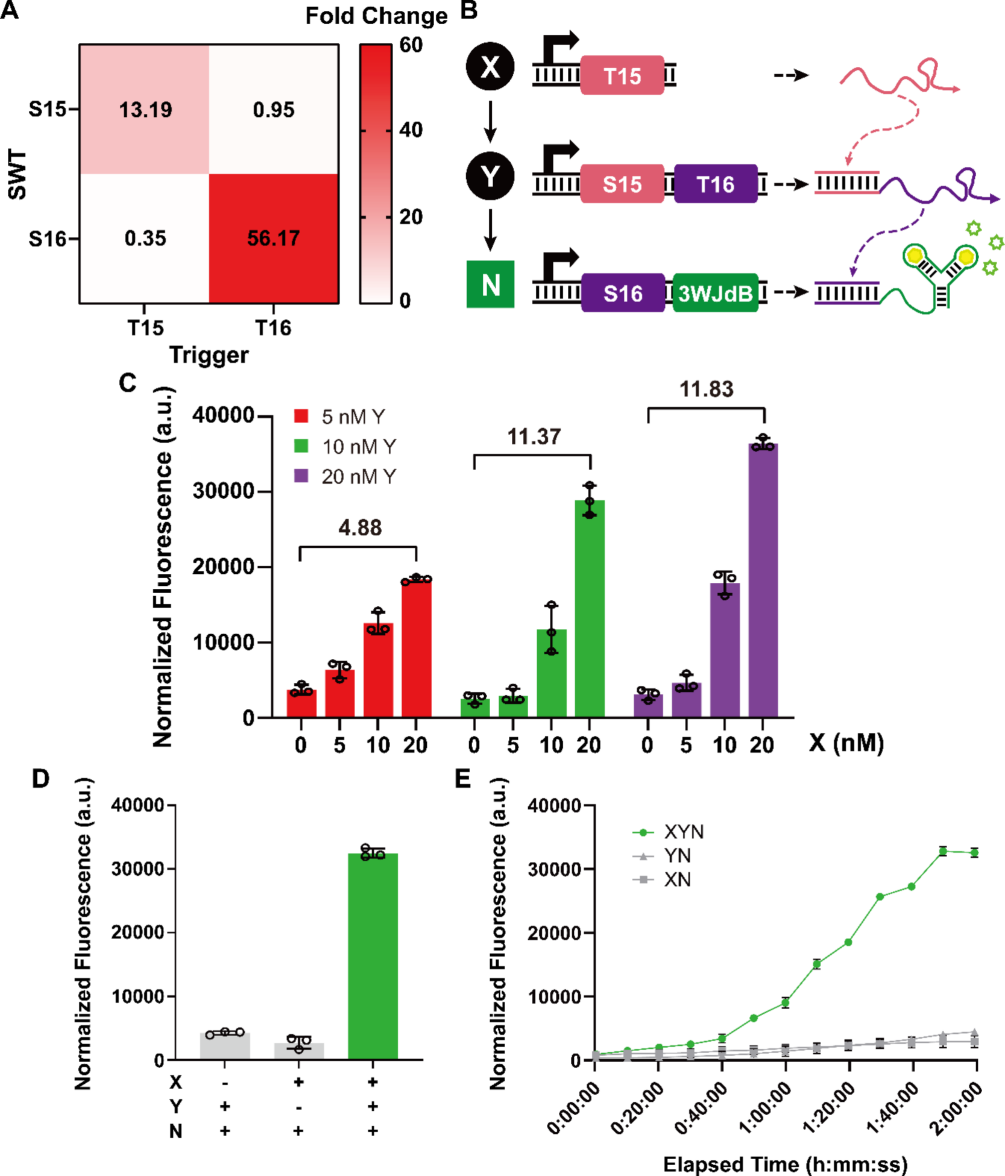
Assessment of SWT orthogonality and a three-layer cascade circuit. (**A**) Orthogonality characterization of S15 and S16. Each element of the matrix represents the ON/OFF ratio for the indicated SWT/trigger combination. Fold change value was represented by a color scale. (**B**) Schematic of a three-layer cascade circuit. The input layer X generates T15, which in turn activates the signal processing layer Y (S15-T16) to express T16, enabling the expression of reporter from the final reporter layer N (S16-3WJdB). (**C**) Characterization of the three-layer cascade circuit. The concentration of the report module N (S16-3WJdB) was fixed at 10 nM, and the concentrations for input X and signal processing module Y were adjusted. (**D**) Combinatorial test of different components for three-layer cascade circuit. (**E**) Time-course measurement for the three-layer cascade circuit. For 3D and 3E, X and Y modules were fixed at 20 nM, and N module was fixed at 10 nM. All data shown are *n* = 3 independent biological replicates. Error bars represent the standard deviation (s.d.) of three biological replicates

In vitro experiments verified the performance of these SWTs with expected dynamic range and good orthogonality (Fig. 3A). Based on these, we constructed a three-layer cascade circuit where T15 forms the first input layer, S15 with T16 as an output forms the second layer, and S16 with 3WJdB forms the third and final layer (Fig. 3B). The input T15 activated S15, resulting in the continued transcription and production of trigger T16, which subsequently activated S16 to transcribe and produce the fluorescence output of 3WJdB. The three-layer cascade circuit produced a maximum of 11.83-fold increase in fluorescence signal (Fig. 3C). The experiments that combine different layer components demonstrated that the orthogonal SWT-based three-layer circuit operated as expected and required the presence of all three components for functionality (Fig. 3D and E).

We also aimed to construct a four-layer cascade using a set of three orthogonal SWTs (Supplementary Figures S12, S13). However, a noticeable crosstalk was observed when the three distinct components of a four-layer cascade were combined indicating that there are further design features that need to be considered and that a large circuit can exacerbate the potential crosstalk and leakages (Supplementary Figure S14). Thus, we need further refinement in design algorithms and experimental procedures to implement higher-order circuits.

### Multilayered logic circuit

**Fig. 4:**
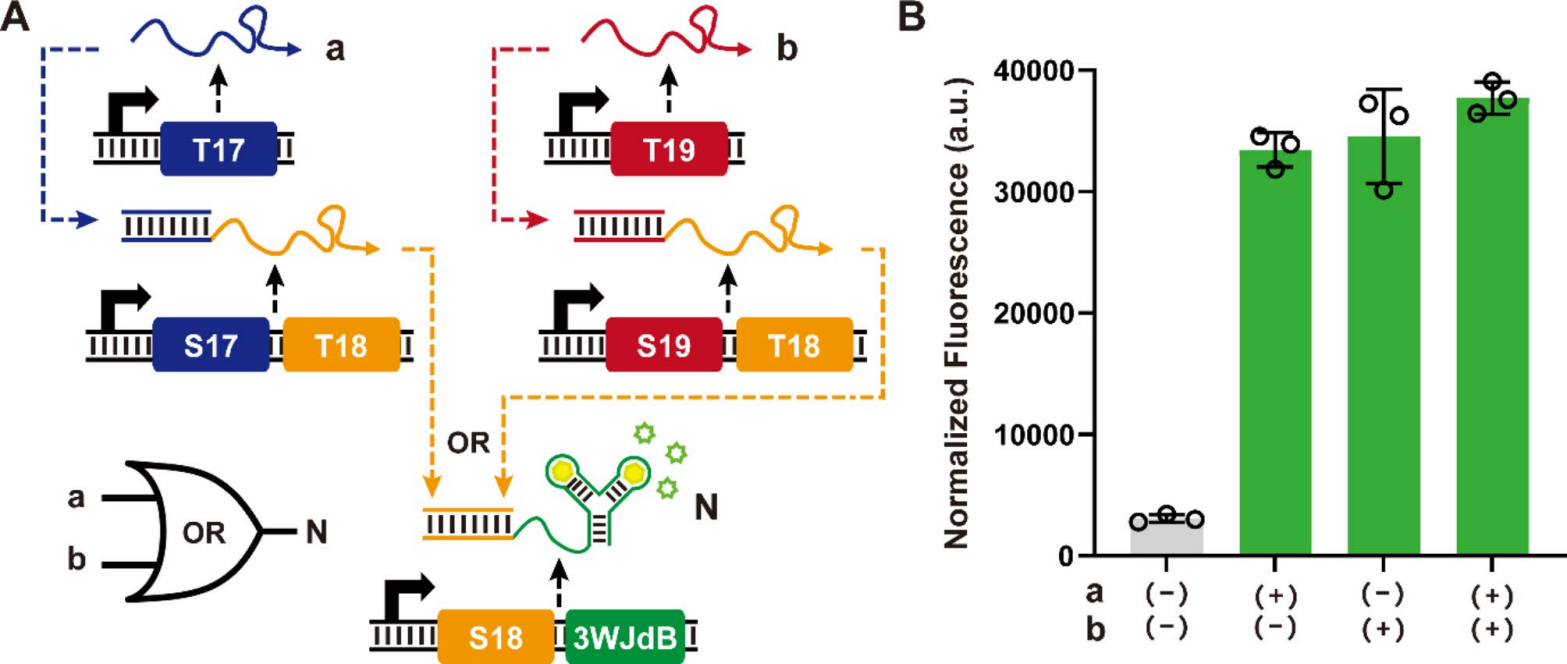
A three-layer cascade OR gate. (**A**) Schematic of a three-layer OR gate. The inputs a (T17) and b (T19) signals are processed by the signal processing layers S17-T18 and S19-T18 for conversion to a common signal T18, which in turn activates the reporter expression in the final reporter layer S18-3WJdB. (**B**) Characterization of the OR gate with different combinations of input signals a and b. All data shown are *n* = 3 independent biological replicates. Error bars represent the standard deviation (s.d.) of three biological replicates

Multilayered synthetic logic circuits are essential elements to integrate signals and process information to ultimately control cell behavior [38–40]. Still, there are limited examples of synthetic RNA-only multilayered logic circuits due in part to stringent requirements on orthogonality, composability, and signal transmission for such circuits. Therefore, we aimed to demonstrate a multilayered logic circuit where the two input signals activate their cognate SWT such that they each can generate a common output, which in turn could be used as an input for the final SWT layer with 3WJdB reporter (Fig. 4A). This three-layer cascade with OR function can be constructed using a set of three orthogonal SWTs to generate output when either of the input signal is present. Experimental results showed that the output signal was high when either of the inputs was present or when both inputs were present, consistent with the desired function of layered OR circuit (Fig. 4B). The successful demonstration of the two-input three-layer OR gate indicates that the orthogonality and composability of SWTs can potentially be utilized to construct a more complex multilayered logic circuits for further applications.

## Discussion

In previous reports, Chappell et al. have successfully constructed RNA regulators with the functions of a NOT gate and an AND gate [26]. In this work, the successful construction of OR gate could complement the earlier works for synthetic logic circuits. Together, these basic logic gates can be combined to construct other logic elements such as OR/NOT gates, XOR gates, and other circuits. In addition, Hong et al. integrated SWT with toehold switches to build an AND gate, demonstrating the potential for merging SWTs with other synthetic regulators to build diverse synthetic logic circuits [27]. With the rapid development of synthetic biology, the demand for a large number of standardized and modular regulatory elements is a growing challenge for the field. Compared to protein regulators, RNA regulators exhibit lower cytotoxicity and shorter reaction times [41, 42], and impose less stress on cells [43], providing a rationale for further application of synthetic RNA regulators including SWTs in synthetic gene circuit construction. The optimizations reported in this work reflect a targeted effort to refine the efficacy of SWTs, including modifications to the toehold regions for enhanced activation and the careful tuning of GC content to achieve a delicate balance between stability and activity. Our design approach emphasizes the modular and scalable nature of SWT components, facilitating the assembly of complex, multi-layered circuits that can be seamlessly integrated into diverse genetic backgrounds.

To increase the complexity of synthetic circuits, we used an automated design algorithm to generate mutually orthogonal sequences and successfully constructed a three-layer cascade and a three-layer OR gate. The introduction of computer algorithms allowed us to pre-screen sequences that meet the orthogonality requirements, greatly reducing the effort in experimental validation steps. Additionally, we have successfully generated up to five mutually orthogonal sequences (Supplementary Figure S15). While we employed the well-characterized T500 terminator for most SWT designs, alternative terminator sequences can be adopted in order to obtain a larger library of orthogonal SWT sequences. In addition, the design algorithm could be improved by taking into account the dynamic folding process, molecular ratio, and cellular burden. At the same time, more experimental data may be needed for machine learning to improve the algorithm [36]. 

RNA synthetic biology is rapidly advancing, providing diverse techniques for gene expression regulation [44–48]. CRISPRi utilizing the CRISPR-Cas system, silences genes by guiding the Cas protein with specific RNAs to the target gene’s nearby DNA sequence [49–51]. In comparison, SWT uses the designed structure of RNA to halt RNA polymerase, achieving targeted transcriptional regulation. CRISPRi has successfully been used to construct NIMPLY logic gates and glucose-detecting bacteria [52, 53]. Recent discovery of CRISPRa has further expanded gene regulatory circuit design [54, 55]. Tickman et al. successfully integrated CRISPRa with existing CRISPRi-based systems, resulting in the construction of complex multilayered CRISPRa/i cascades and feedforward loops [56]. In addition, Cas13a, a novel CRISPR RNA-guided RNA-targeting effector, suppresses mRNA expression with high specificity [57–60]. Cas13a enables RNA-based gene regulatory networks, offering an alternative to direct editing of genome with its associated risks [61]. Because the input and output of SWTs are both RNAs and synthetic circuits with SWT elements can control the expression of guide RNA sequences in a straightforward manner, SWT can be utilized in conjunction with these powerful CRISPR regulators to enrich gene regulation methods and expand gene circuit architecture.

## Conclusions

In this study, we explored the design strategy of SWT, including a new set of sequences for transcription terminators to construct high-performance SWTs. These SWTs have expanded the repertoire of RNA-based transcriptional regulators, providing valuable references for designing orthogonal circuits and enriching the toolbox of synthetic biology. To enhance the orthogonality of SWT library, we developed an algorithm capable of generating a set of orthogonal sequences. Using SWTs designed by the algorithm, we constructed a three-layer cascade circuit and a two-input three-layer OR gate. The successful implementation of multilayered gene circuits indicates that SWT demonstrated the potential to expand the complexity of synthetic gene circuits. Our work presents the exploration and development of SWT for constructing complex multilayered gene circuits. We highlight the potential of de-novo-designed synthetic transcription terminators as genetic parts in circuit construction and emphasize the design flexibility enabled by advanced nucleic acid sequence design tools. Together, these findings provide a new set of tools to expand the suite of high-performance regulatory elements for synthetic biology, paving the way for the streamlined construction of complex synthetic gene circuits in the future.

## Electronic Supplementary Material

Below is the link to the electronic supplementary material.

Supplementary Material 1: Additional file 1 Description of data: Detailed experimental procedures for design algorithm, supplementary figures for secondary structure simulations of SWTs, characterizations of the SWTs at different concentrations, crosstalk simulations between SWTs, orthogonality assessments of SWTs, supplementary tables for construct sequences used in this study.

## Data Availability

All data generated or analyzed during this study are included in this article and its supplementary material files, or available from the corresponding author on reasonable request.

## Abbreviations

STAR: Small Transcription Activating RNA
SWT: Switchable transcription terminator
E. coli: Escherichia coli
3WJdB: 3-Way Junction dimeric Broccoli
