# Supplementary material for: Construction of multilayered gene circuits using de-novo-designed synthetic transcriptional regulators in cell-free systems
Source: J Biol Eng. 2024 Nov 5;18:64. doi: 10.1186/s13036-024-00459-8 (PMC11539451; doi:10.1186/s13036-024-00459-8)
Supplement: Supplementary file 1 — Supplementary Material 1: Additional file 1 Description of data: Detailed experimental procedures for design algorithm, supplementary figures for secondary structure simulations of SWTs, characterizations of the SWTs at different concentrations, crosstalk simulations between SWTs, orthogonality assessments of SWTs, supplementary tables for construct sequences used in this study. [file 13036_2024_459_MOESM1_ESM.docx]

**Supplementary Material**

**for ”Construction of multilayered gene circuits using de-novo-designed synthetic transcriptional regulators in cell-free systems”**

Mingming Zhao^1,†^, Jeongwon Kim^2,†^, Jiayan Jiao^1^, Yelin Lim^2^, Xianai Shi^1,3,4^, Shaobin Guo^1,3,4,*^ and Jongmin Kim^2,*^

^1^ College of Biological Science and Engineering, Fuzhou University, Fuzhou 350108, Fujian, China

^2^ Department of Life Sciences, Pohang University of Science and Technology, Pohang 37673, Korea

^3^ Fujian Key Laboratory of Medical Instrument and Pharmaceutical Technology, Fuzhou University, 350108 Fuzhou, Fujian, China

^4^ International Joint Laboratory of Intelligent Health Care, Fuzhou University, 350108 Fuzhou, Fujian, China

^*^ Corresponding author(s). E-mail(s): sguo@fzu.edu.cn; jongmin.kim@postech.ac.kr

^†^ These authors contributed equally to this work.

**Contents**

[**Experimental Procedures - The design of algorithm 2**](#_Toc20624)

[Strategy for algorithm design 2](#_Toc22762)

[General simulation conditions 2](#_Toc31809)

[Sequence and structure specification 2](#_Toc11338)

[Test-tube setting 3](#_Toc23604)

[Sample code 3](#_Toc8660)

[**Supplementary Figures 6**](#_Toc20624)

[Supplementary Figure S1. Schematic of the SWT design motif for RNA-RNA interaction. 6](#_Toc1657)

[Supplementary Figure S2. Selection of trigger RNAs. 7](#_Toc1657)

[Supplementary Figure S3. Secondary structure simulation of SWTs. 8](#_Toc1657)

[Supplementary Figure S4. Characterization of the SWTs at different concentrations. 9](#_Toc7060)

[Supplementary Figure S5. Leakage of SWTs with different toehold GC contents. 11](#_Toc47)

[Supplementary Figure S6. Time-course measurement for single SWT.](#_Toc1435) 11

[Supplementary Figure S7. Characterization and Structural Simulation of S13.](#_Toc47) 12

[Supplementary Figure S8. Crosstalk simulation of S1 and S14.](#_Toc19791) 13

[Supplementary Figure S9. Crosstalk simulation of S14 and T1.](#_Toc263) 14

[Supplementary Figure S10. Orthogonal assessment and characterization of S14.](#_Toc12484) 15

[Supplementary Figure S11. Crosstalk simulation of S15 and S16.](#_Toc11345) 16

[Supplementary Figure S12. Crosstalk simulation between S17, S18 and S19.](#_Toc1435) 17

[Supplementary Figure S13. Assessment of SWT orthogonality and a four-layer cascade circuit.](#_Toc1435) 18

[Supplementary Figure S14. Layer control experiments for four-layer cascade circuit.](#_Toc1435) 19

[Supplementary Figure S15. Orthogonality testing of five mutually orthogonal sequences.](#_Toc1435) 20

[**Supplementary Tables...........................................................................................................................**](#_Toc20624)**21**

[Supplementary Table S1. Examples of DNA plasmid sequences...................................................](#_Toc16573)21

[Supplementary Table S2. Sensor sequences used in this study.](#_Toc26837) 28

[Supplementary Table S3. Trigger sequences used in this study.](#_Toc865) 29

[**REFERENCES**](#_Toc25967) **30**

**Experimental Procedures – The design of algorithm**

This section describes the detailed specifications of algorithm for orthogonal SWT and trigger set generation.

**Strategy for algorithm design**

Conventional in silico sequence design utilized "*complex*" design which can be defined as an optimization problem of *complex* ensemble defect.[1] However, since the *complex* ensemble defect only evaluates the incorrectly paired nucleotides of the specific complex at equilibrium state, additional scoring methods and/or assessment algorithm were required to compute orthogonality between diverse sets of sequences. In order to reduce those computational effort and generate orthogonal sequences directly during the design process, we utilized "*multi-tube*" design method for our designing algorithm.[2] Unlike *complex* ensemble defect, *test tube* ensemble defect is defined with not only the target complex ensemble, but also the concentration of target complex and "off-target" complex ensemble (which concentration is vanished as 0) in virtual reaction test tube.[3] Therefore, in *multi-tube* design, it is possible to design orthogonal sequence sets that takes into account both the interaction between corresponding SWT set (target complex) and the other unwanted interactions (off-target complex).

**General simulation conditions**

We built designing algorithm based on NUPACK Python module (NUPACK 4.0) which includes libraries for multi-tube sequence design.[4] For consistency with the previous SWT characterization results, ‘rna99-nupack3’ parameter was applied for free-energy calculation and the temperature was set to 37 ℃. To preclude improper cases where the repetation of the same bases or pairings appear, we applied pattern prevention of following cases for unspecified sequences: ‘AAA’, ‘CCC’, ‘GGG’, ‘UUU’, ‘MMMM’, ‘KKKK’, ’WWWW’, ‘SSSS’, ‘RRRR’, ‘YYYY’.

**Sequence and structure specification**

For each SWT, the stem-loop region was conserved as T500 terminator sequence and the toehold region was set as random sequence of 40 bases. The target structure of SWT was defined as a completely linear toehold region followed by the T500 terminator structure predicted by the NUPACK web application. Following the characterization results, GC-content of the toehold region was set to be 50~60 %. For the trigger RNA, we initially defined it as 40 base sequences which complementary to the toehold region of the corresponding SWT to reduce calculation time. However, in vitro tests using those sequences showed that orthogonality was not well secured. Therefore, we changed it to include stem sequences of T500, which is more likely to the trigger RNA in practical experimental conditions. The structure of trigger RNA was defined to be completely linear, and fully combined structure between the SWT and the corresponding trigger was set as the target complex structure. All the other complexes and structures not defined above were categorized as off-target.

**Test-tube setting**

To implement multi-tube design based orthogonal algorithm, we defined three types of test tubes: 1) test tubes containing individual constructs to check unwanted self-assembly of each construct, 2) test tubes containing the entire SWTs or the entire trigger RNAs to check unwanted interaction between among SWTs or trigger RNAs, and 3) test tubes containing SWT and trigger RNA pairs to check ‘orthogonality’. Following the principle, total 10 test tubes for 2-orthogonal pairs and 17 test tubes for 3-orthogonal pairs were set. The weight parameter for each tube was optimized through iterative sequence generation and structure prediction. The concentration of each construct and their target complex followed the conditions of in vitro experiment.

**Sample code**

from nupack import *

config.parallelism = True

RNA_model = Model(material='rna99-nupack3', celsius=37)

toe_a = Domain('N40', name='toe_a')

toe_b = Domain('N40', name='toe_b')

t500 = Domain('AAAGCCCGCCGAAAGGCGGGCUUUUUUUU', name='t500')

t500_stem = Domain('GGCGGGCUUU', name='t500_stem')

swt_a = TargetStrand([toe_a, t500], name='swt_a')

swt_b = TargetStrand([toe_b, t500], name='swt_b')

tr_a = TargetStrand([t500_stem, ~toe_a], name='tr_a')

tr_b = TargetStrand([t500_stem, ~toe_b], name='tr_b')

SWT_A = TargetComplex([swt_a], '........................................((((((((((....)))))))))).....', name='SWT_A')

SWT_B = TargetComplex([swt_b], '........................................((((((((((....)))))))))).....', name='SWT_B')

TR_A = TargetComplex([tr_a], '..................................................', name='TR_A')

TR_B = TargetComplex([tr_b], '..................................................', name='TR_B')

SWT_TR_AA = TargetComplex([swt_a, tr_a], '((((((((((((((((((((((((((((((((((((((((((((((((((...................+))))))))))))))))))))))))))))))))))))))))))))))))))', name='SWT_TR_AA')

SWT_TR_BB = TargetComplex([swt_b, tr_b], '((((((((((((((((((((((((((((((((((((((((((((((((((...................+))))))))))))))))))))))))))))))))))))))))))))))))))', name='SWT_TR_BB')

pattern_restriction = Pattern(['A3', 'C3', 'G3', 'U3', 'M4', 'K4', 'W4', 'S4', 'R4', 'Y4'], scope = [toe_a, toe_b])

simA = Similarity([toe_a], 'S40', limits=[0.50, 0.60])

simB = Similarity([toe_b], 'S40', limits=[0.50, 0.60])

tube_SWT_A = TargetTube(on_targets = {SWT_A: 1e-8}, off_targets = SetSpec(max_size=2), name = 'tube_SWT_A')

tube_SWT_B = TargetTube(on_targets = {SWT_B: 1e-8}, off_targets = SetSpec(max_size=2), name = 'tube_SWT_B')

tube_TR_A = TargetTube(on_targets = {TR_A: 1e-8}, off_targets = SetSpec(max_size=2), name = 'tube_TR_A')

tube_TR_B = TargetTube(on_targets = {TR_B: 1e-8}, off_targets = SetSpec(max_size=2), name = 'tube_TR_B')

tube_SWTs = TargetTube(on_targets = {SWT_A: 1e-8, SWT_B: 1e-8}, off_targets = SetSpec(max_size=2), name = 'tube_SWTs')

tube_TRs = TargetTube(on_targets = {TR_A: 1e-8, TR_B: 1e-8}, off_targets = SetSpec(max_size=2), name = 'tube_TRs')

tube_AA = TargetTube(on_targets = {SWT_TR_AA: 1e-8}, off_targets = SetSpec(max_size=2), name = 'tube_AA')

tube_AB = TargetTube(on_targets = {SWT_A: 1e-8, TR_B: 1e-8}, off_targets = SetSpec(max_size=2), name = 'tube_AB')

tube_BA = TargetTube(on_targets = {SWT_B: 1e-8, TR_A: 1e-8}, off_targets = SetSpec(max_size=2), name = 'tube_BA')

tube_BB = TargetTube(on_targets = {SWT_TR_BB: 1e-8}, off_targets = SetSpec(max_size=2), name = 'tube_BB')

my_tubes = [tube_SWT_A, tube_SWT_B, tube_TR_A, tube_TR_B, tube_SWTs, tube_TRs,

tube_AA, tube_AB, tube_BA, tube_BB]

weights = Weights(my_tubes)

weights[:, :, :, tube_SWT_A] = 3000

weights[:, :, :, tube_SWT_B] = 3000

weights[:, :, :, tube_SWTs] = 300

weights[:, :, :, tube_TRs] = 600

weights[:, :, :, tube_AB] = 800

weights[:, :, :, tube_BA] = 800

my_design = tube_design(tubes=my_tubes, model=RNA_model, hard_constraints=[pattern_restriction, simA, simB], defect_weights=weights)

my_jobs = my_design.launch(trials=1)

my_jobs.wait()

my_results = my_jobs.final_results()

print(my_results[0])

**Supplemetary Figures**


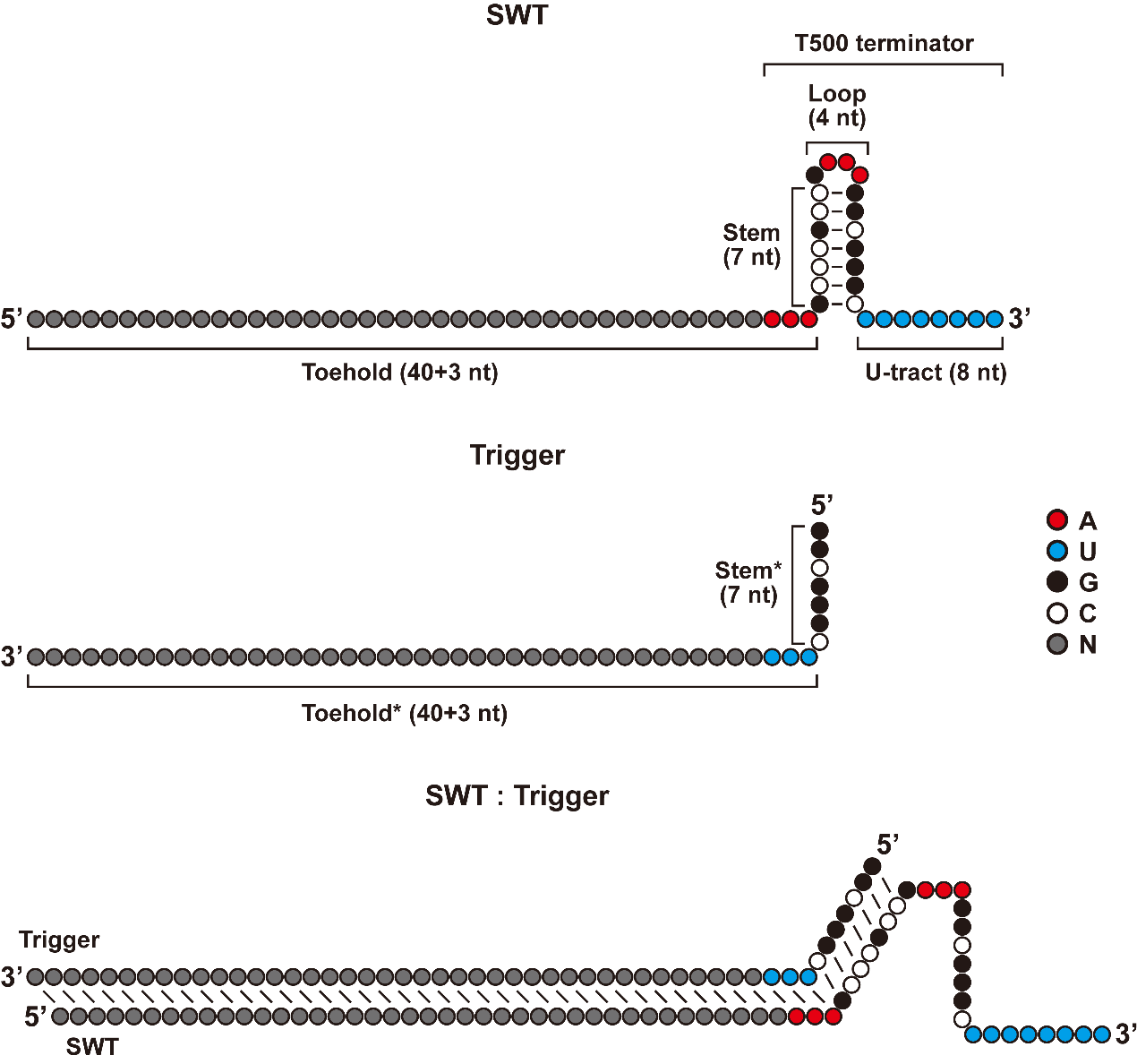


**Supplementary Figure S1. Schematic of the SWT design motif for RNA-RNA interaction.**

Schematic of the sequence and structure constraints for the SWT, Trigger RNA, and SWT-Trigger complex. Nucleotides colored according to identity with N representing an unconstrained nucleotide.


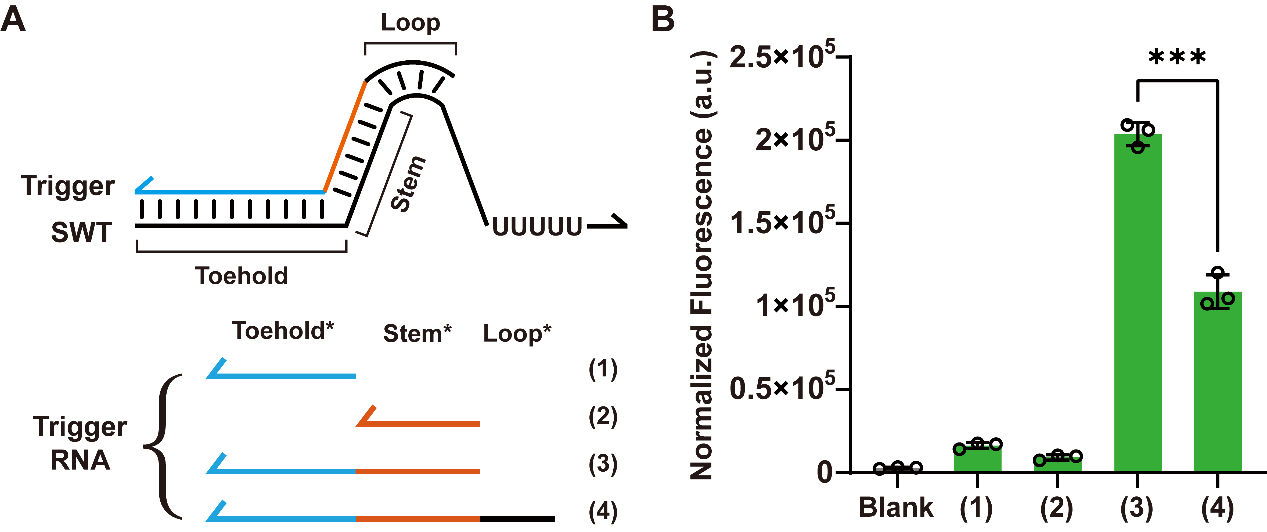


**Supplementary Figure S2. Selection of trigger RNAs.**

(A) Different designs of trigger RNAs. (1-4) represent triggers designed with four different design choices, and asterisks represent the complementary sequence of the corresponding domain of SWT. (B) The influence of different trigger designs on the activation value of SWT. Triggers are composed of complementary sequences of SWT. All data shown are n = 3 independent biological replicates. For data in A and B, Welch’s t-tests were performed on each construct; ***P < 0.001 indicates the change of normalized fluorescence is statistically significantly different. Error bars represent the standard deviation (s.d.) of three biological replicates.


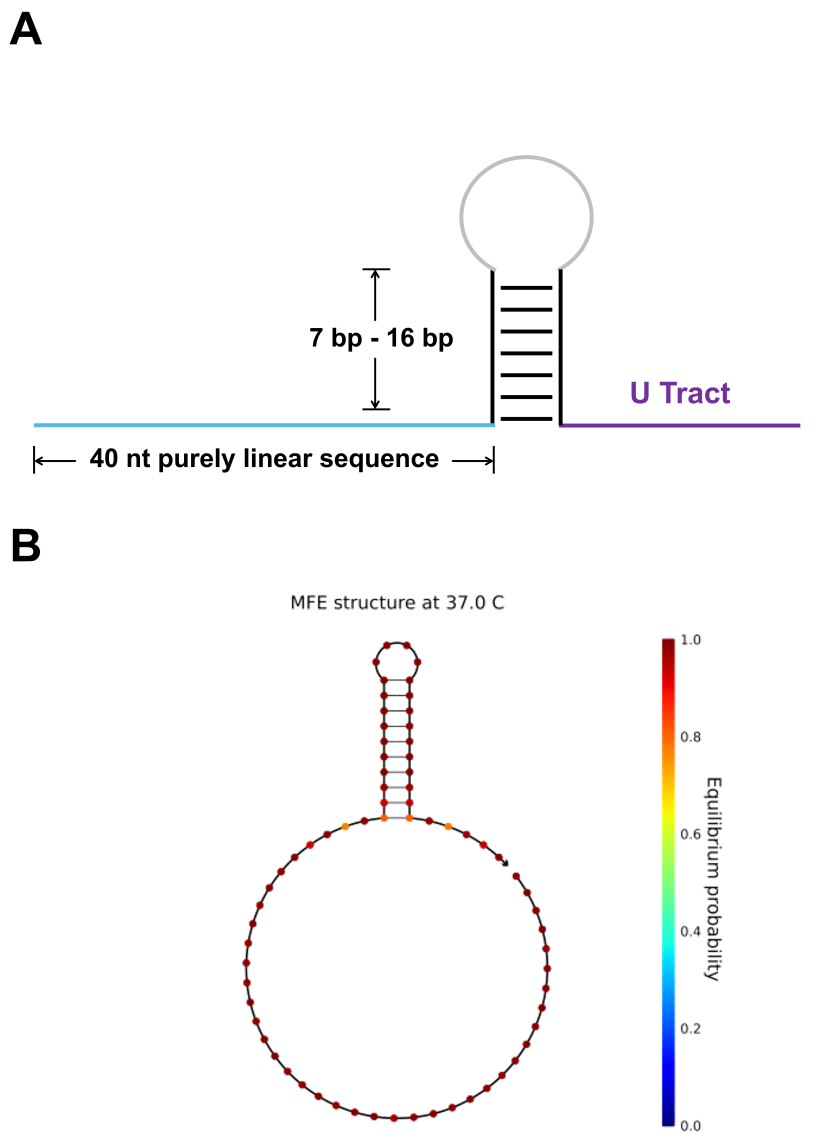


**Supplementary Figure S3. Secondary structure simulation of SWTs.**

(A) Schematic diagram of the structure of SWT. SWT includes a 40 nt purely linear toehold sequence (Blue), stem sequences ranging from 7 to 16 nt (Black), and an 8 nt U tract (Purple). When designing SWT, it is necessary to strictly ensure that the structure of the toehold region is purely linear. It should be noted that although the secondary structure of the 40 nt toehold region can maintain a purely linear structure when simulated alone, the structure of the toehold region may change when combined with the terminator structure. Therefore, in most cases, we recommend simulating the secondary structure of the random linear toehold region again after combining it with the termination structure to ensure the consistency of the final structure. (B) Simulation diagram of SWT in NUPACK. During the design, it was required that the simulation diagram of each SWT in NUPACK must match the structure shown in here.


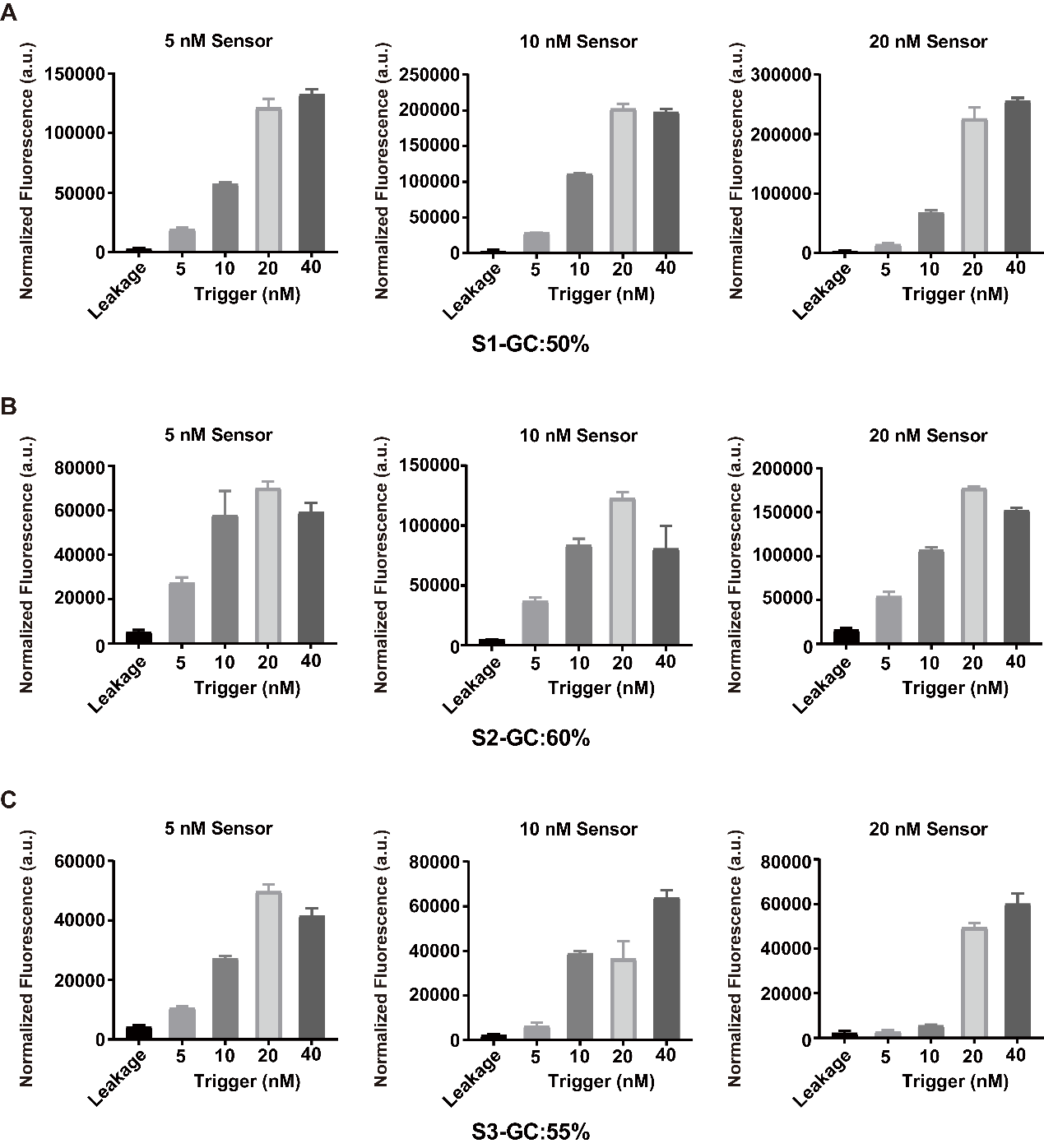


*(continued)*

**
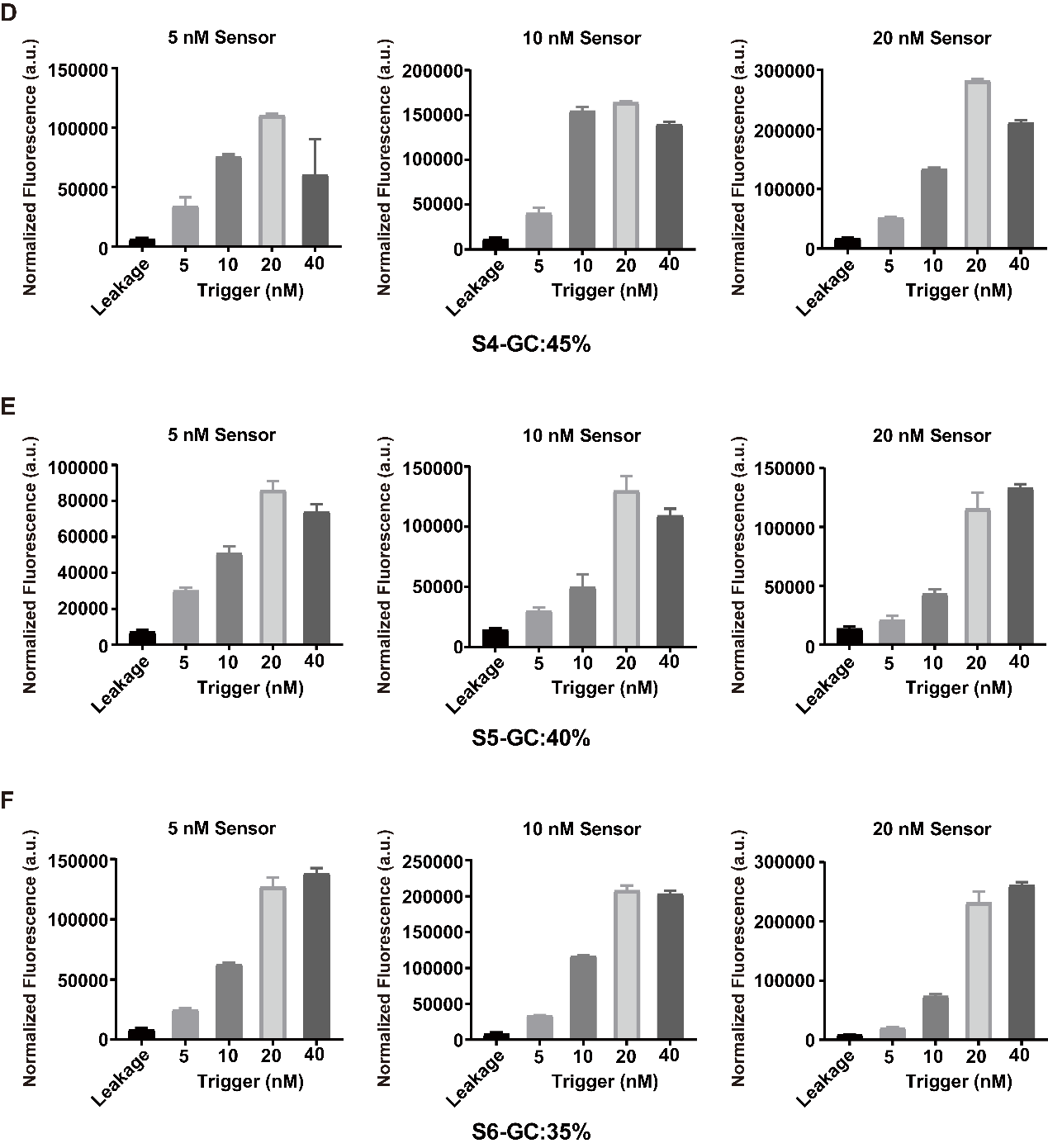
**

**Supplementary Figure S4. Characterization of the SWTs at different concentrations.**

Left, middle, and right panel represent the fluorescence characterization of SWT at concentrations of 5 nM (left), 10 nM (middle), and 20 nM (right), respectively. (A) Sensor S1 (GC-content: 50%). (B) Sensor S2 (GC-content: 60%). (C) Sensor S3 (GC-content: 55%). (D) Sensor S4 (GC-content: 45%). (E) Sensor S5 (GC-content: 40%). (F) Sensor S6 (GC-content: 35%). All data shown are n = 3 independent biological replicates. Error bars indicate the average value of three independent biological replicates ± s.d.


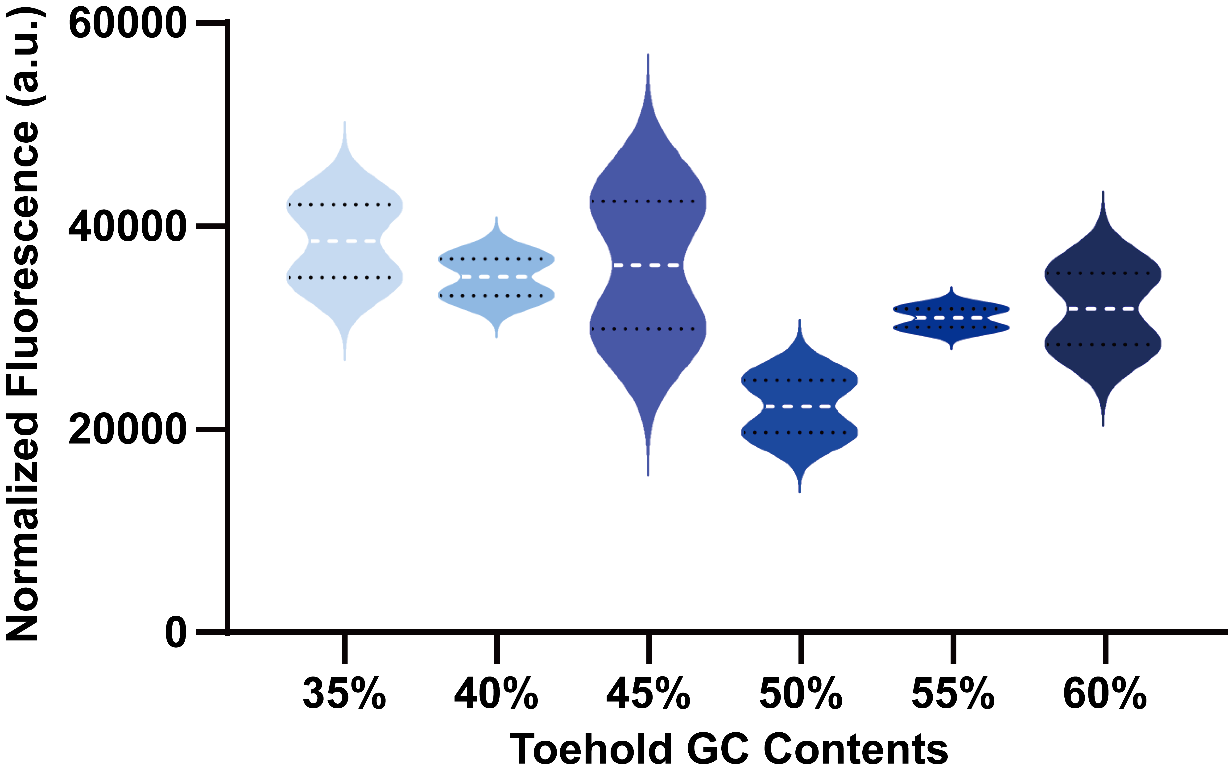


**Supplementary Figure S5. Leakage of SWTs with different toehold GC contents.**

Normalized leakage fluorescence level (without trigger RNA) of diverse SWT constructs with different toehold GC contetns. The leaky expression levels were measured with templates for SWTs at 5 nM.


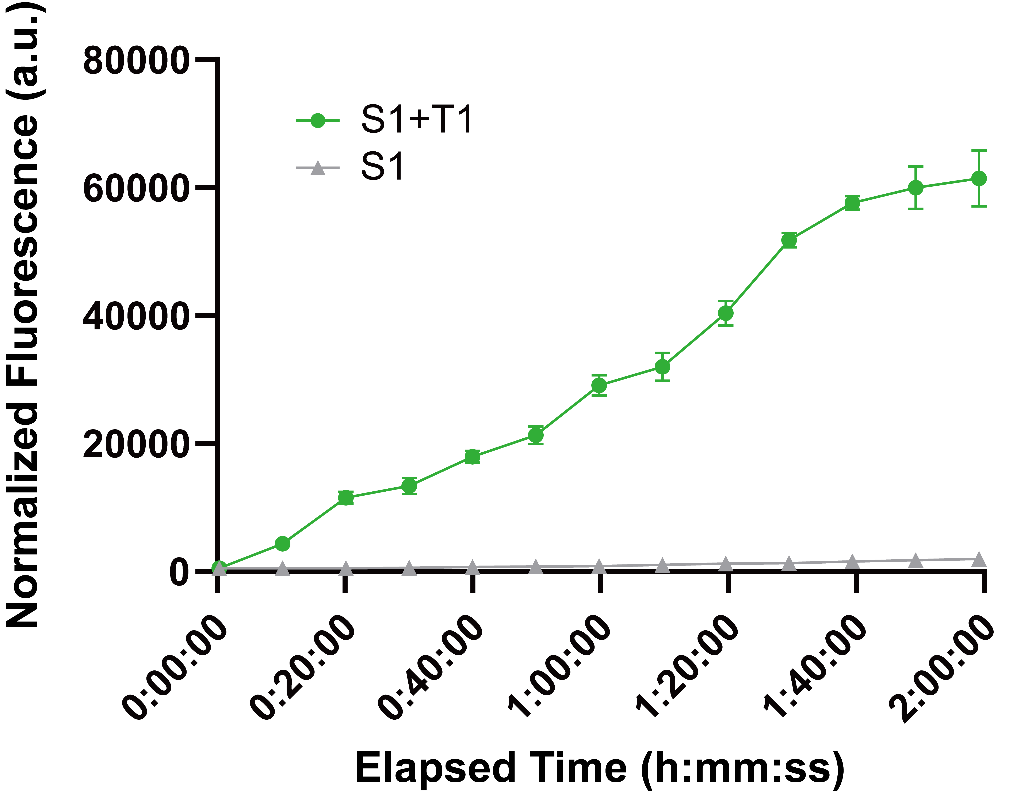


**Supplementary Figure S6. Time-course measurement for single SWT.**

Time-course measurement for single SWT (S1). Fluorescence was measured at 10 min interval and normalized with background reaction (blank). Switch module S1 and trigger module T1 were set to 10 nM.


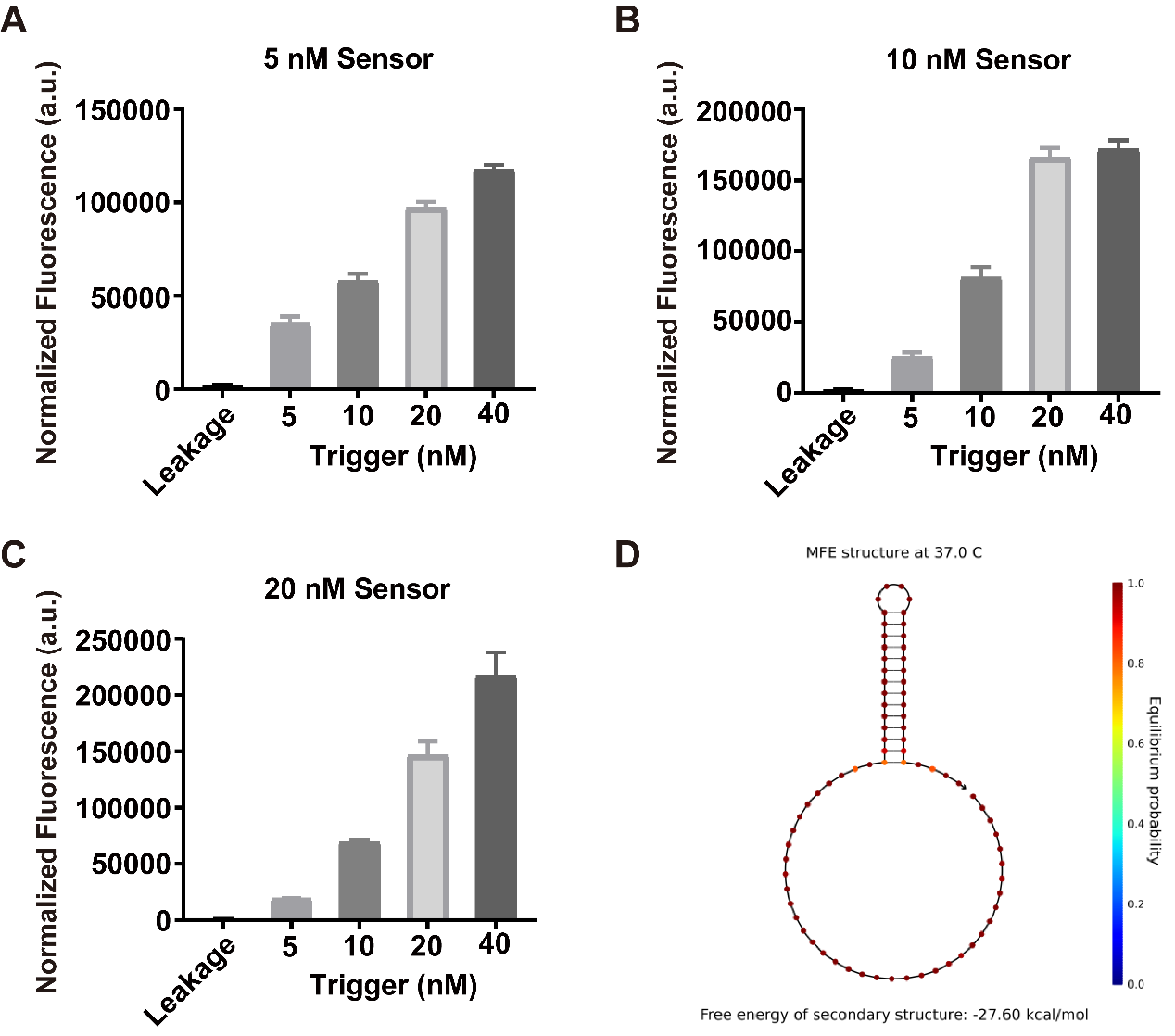


**Supplementary Figure S7. Characterization and structural simulation of S13.**

(A-C) Characterization of the S13 at different concentrations. Error bars indicate the average value of three independent biological replicates ± s.d. (D) Secondary structure simulation of S13.


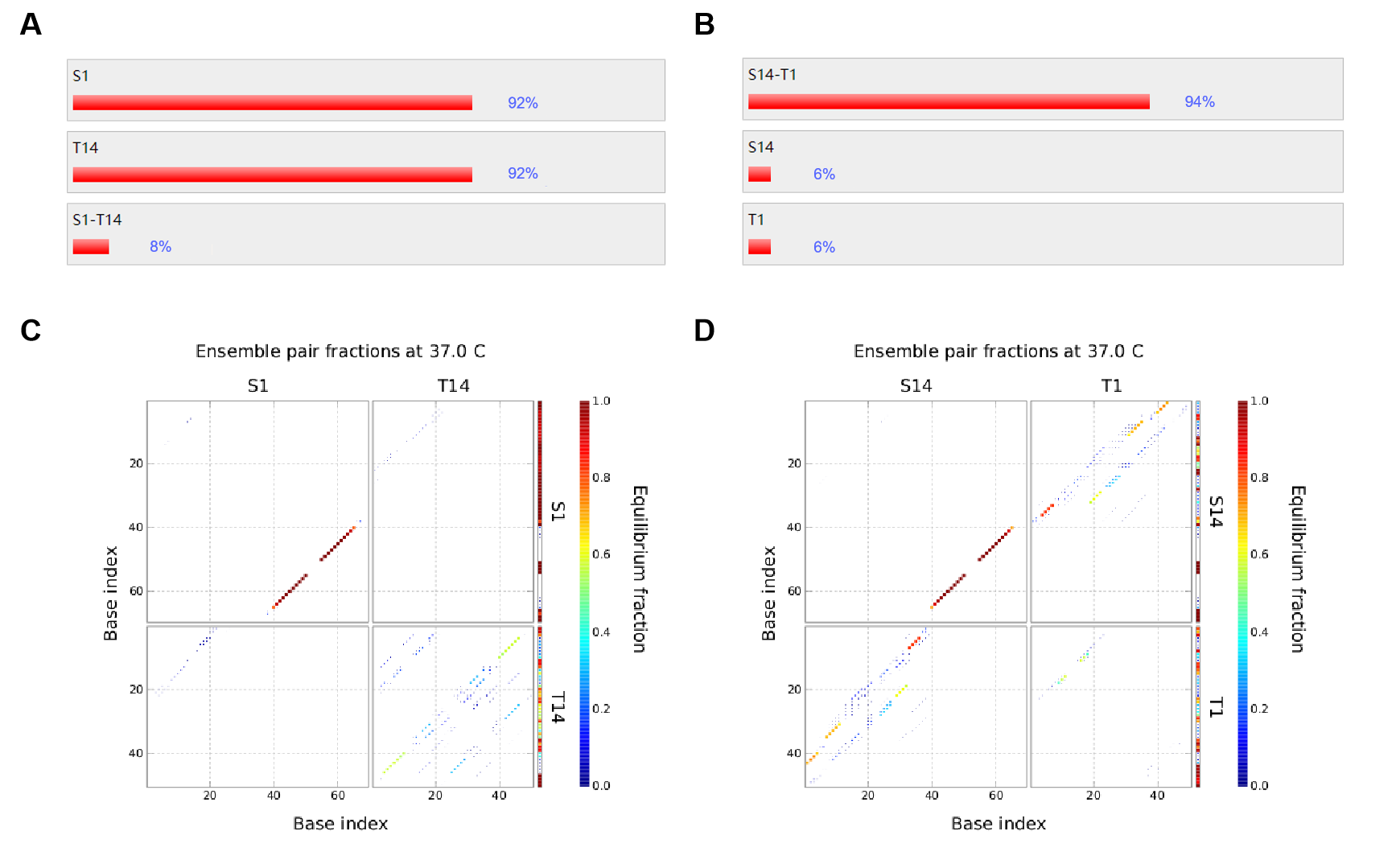


**Supplementary Figure S8. Crosstalk simulation of S1 and S14.**

(A-B) Equilibrium percentage of S1 and S14. The numerical value of the percentage is only kept as an integer. (C-D) Ensemble pair fraction plot of S1 and S14. Depicts equilibrium base-pairing information for the dilute solution, taking into account the equilibrium concentration and base-pairing properties of each ordered complex. The area and color of the dot at row i and column j scale with the equilibrium fraction of base i that is paired with base j in solution (fractions below 0.001 are not depicted). With this convention, the plot can be asymmetric. The area and color of the dot in row i in the column at right scale with the equilibrium fraction of base i that is unpaired. The simulation for crosstalk was set as follows: temperature: 37℃, concentration: 10 nM, maximum complex size: 2.


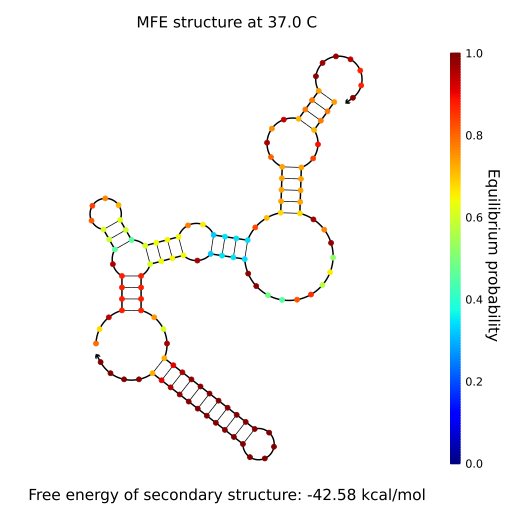


**Supplementary Figure S9. Crosstalk simulation of S14 and T1.**

Simulation diagram of the structure at equilibrium for the crosstalk between S14 and T1.


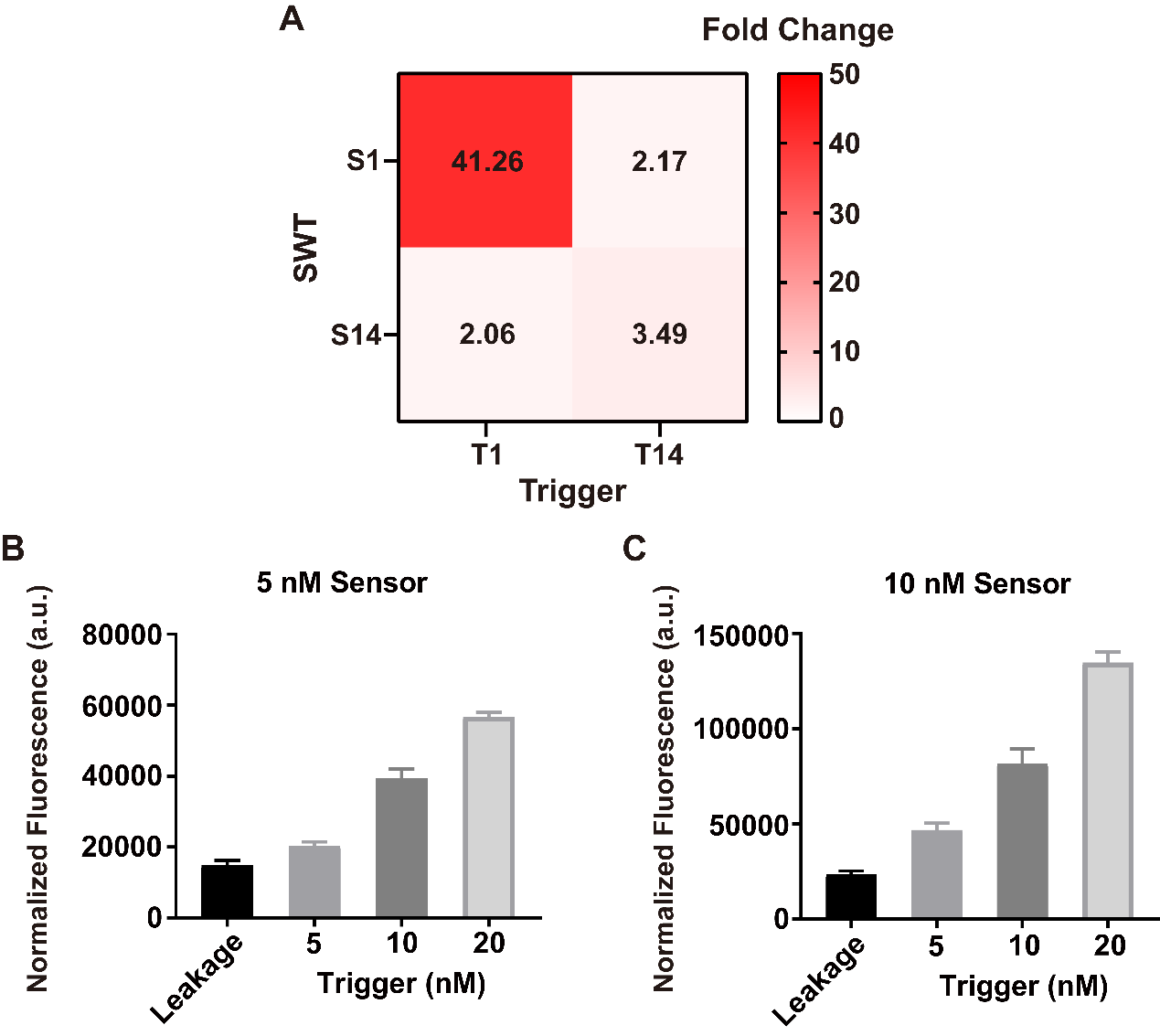


**Supplementary Figure S10. Orthogonality assessment and characterization of S14.**

(A) Orthogonal characterization of S1 and S14. (B-C) Characterization of S14 at different concentrations. The GC content of toehold region in S14 is 40%, resulting in a higher leakage value. Error bars indicate the average value of three independent biological replicates ± s.d.


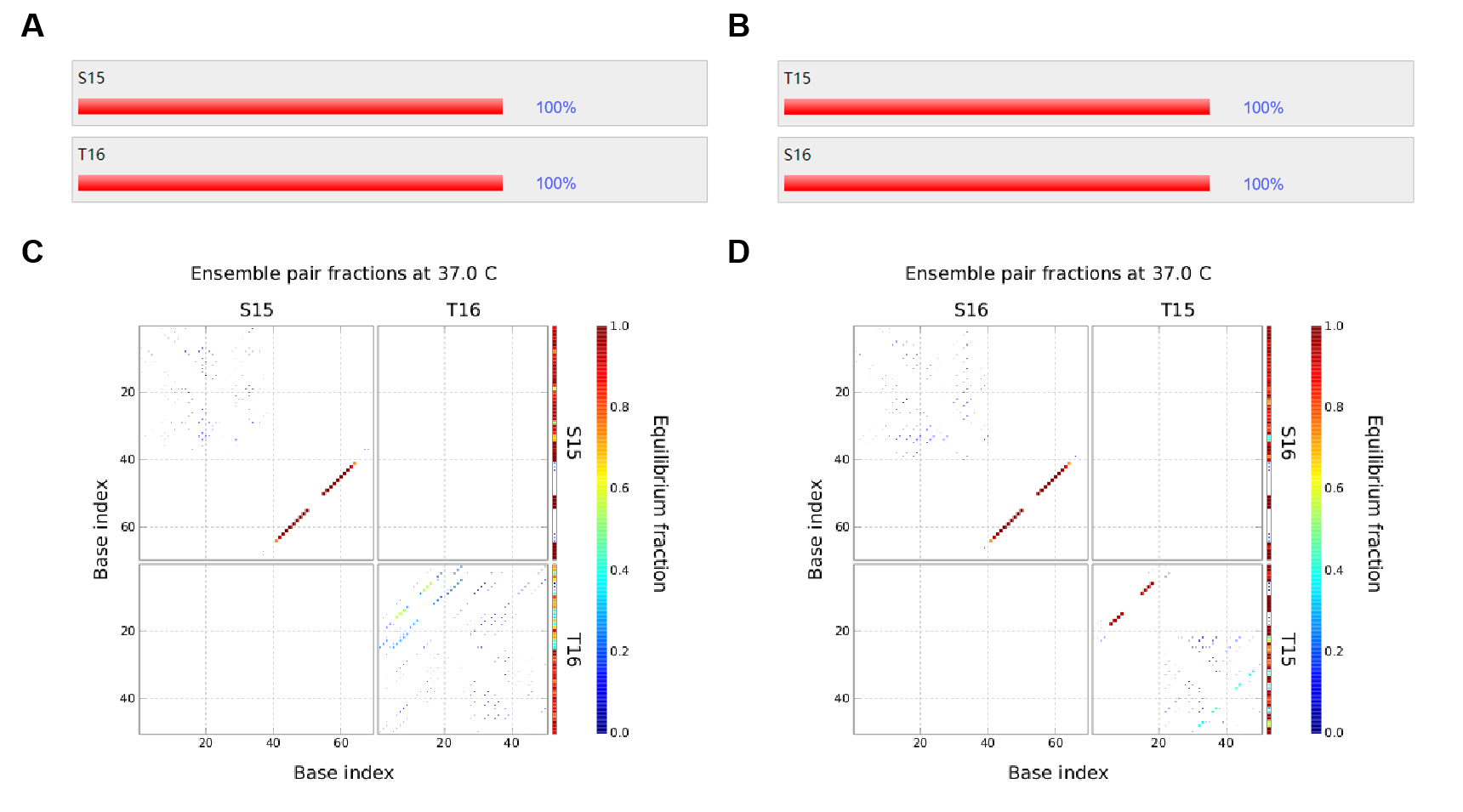


**Supplementary Figure S11. Crosstalk simulation of S15 and S16.**

(A-B) Equilibrium percentage of S1 and S14. The numerical value of the percentage is only kept as an integer. (C-D) Ensemble pair fraction plot of S1 and S14. The simulation for crosstalk was set as follows: temperature: 37 ℃, concentration: 10 nM, maximum complex size: 2

**
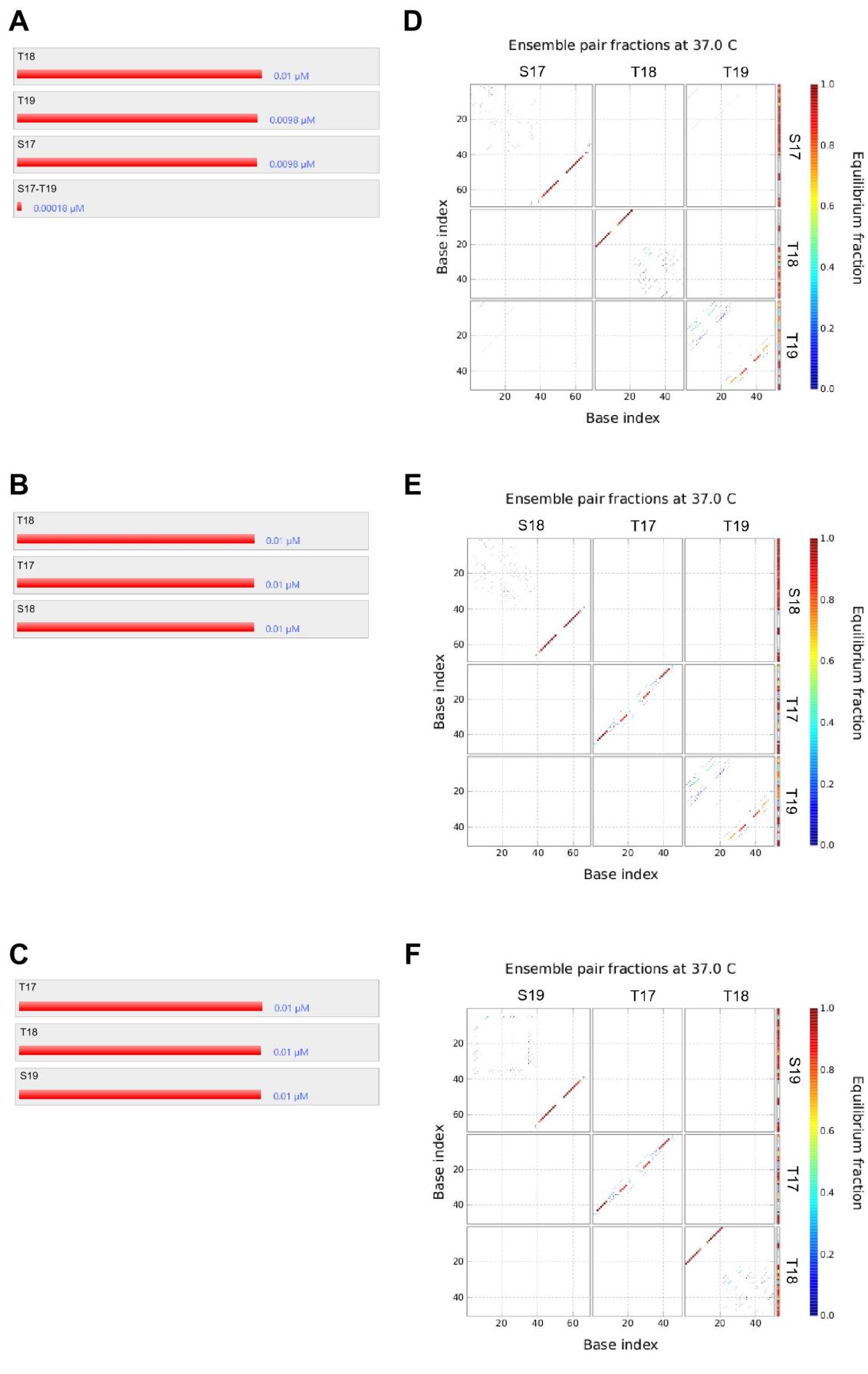
**

**Supplementary Figure S12. Crosstalk simulation between S17, S18, and S19.**

(A-C) Equilibrium percentage of S17, S18, and S19. The numerical value of the percentage is only kept as an integer.(D-F) Ensemble pair fraction plot of S17, S18, and S19. The simulation for crosstalk was set as follows: temperature: 37 ℃, concentration: 10 nM, maximum complex size: 2.


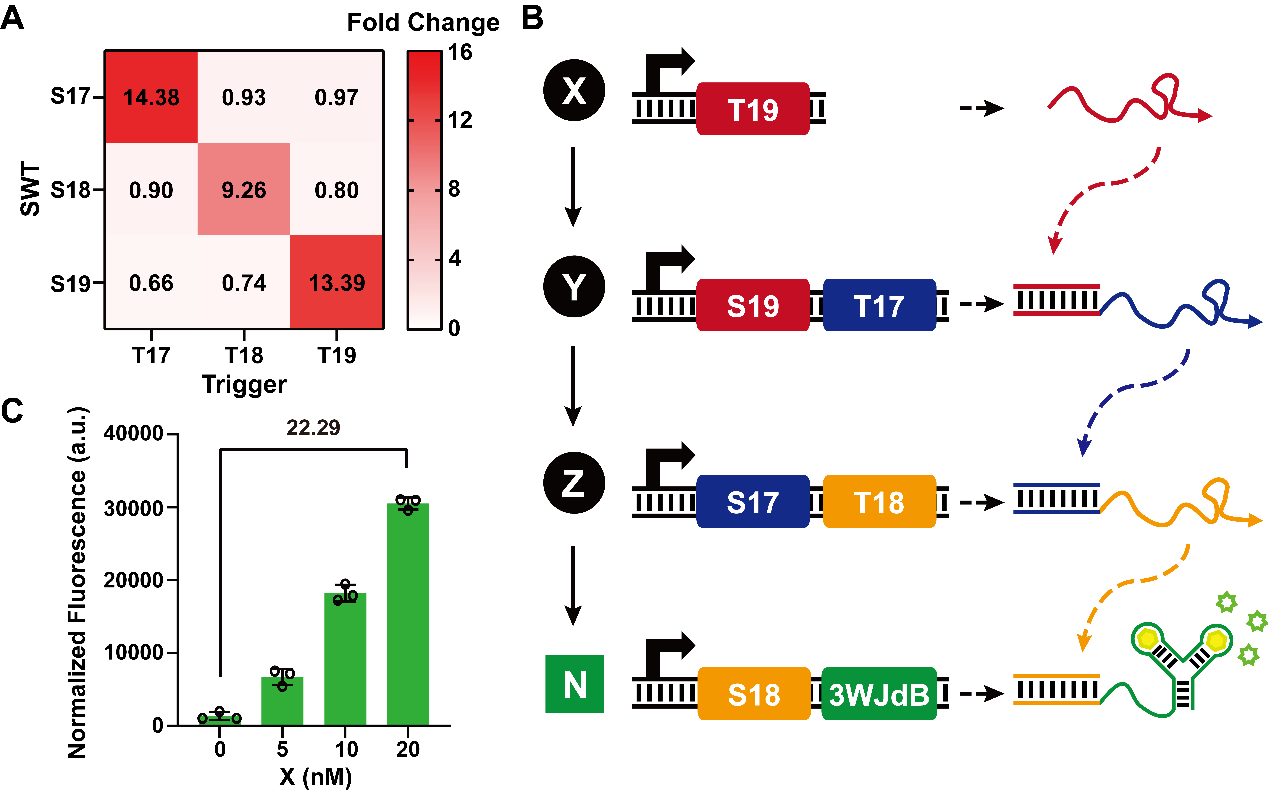


**Supplementary Figure S13. Assessment of SWT orthogonality and a four-layer cascade circuit.**

(A) In vitro experimental characterization of the three orthogonal SWTs generated by the optimized algorithm. (B) Schematic of a four-layer cascade circuit. X (T19) is the input layer, Y (S19-T17) and Z (S17-T18) are signal processing layers and N (S18-3WJdB) is the final report layer. The input signal T19 triggers T17 through Y, then T17 triggers T18 through Z, and finally T18 activates S18 switch in N to express the fluorescence output. (C) Characterization of the four-layer cascade circuit. The concentrations of the report module N and two signal processing modules Y and Z were set to 10 nM. The concentration of input layer X (T19) were tuned to explore the response of final reporters of the four-layer cascade circuit. All data shown are n = 3 independent biological replicates. Error bars indicate the average value of three independent biological replicates ± s.d.


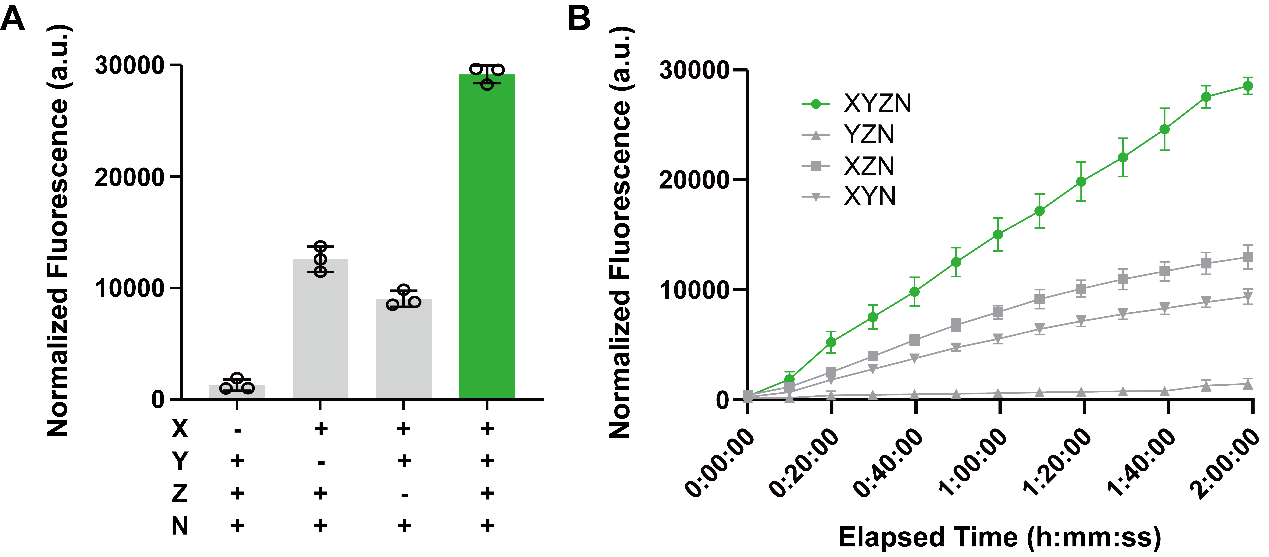


**Supplementary Figure S14. Layer control experiments for four-layer cascade circuit.**

(A) Layer control experiments for four-layer cascade circuit (refer Supplementary Figure S13B) without and with a constant concentration of each module. (B) Time-course measurement for four-layer cascade circuit. X module was set to 20 nM, while Y, Z, and N modules were set to 10 nM.


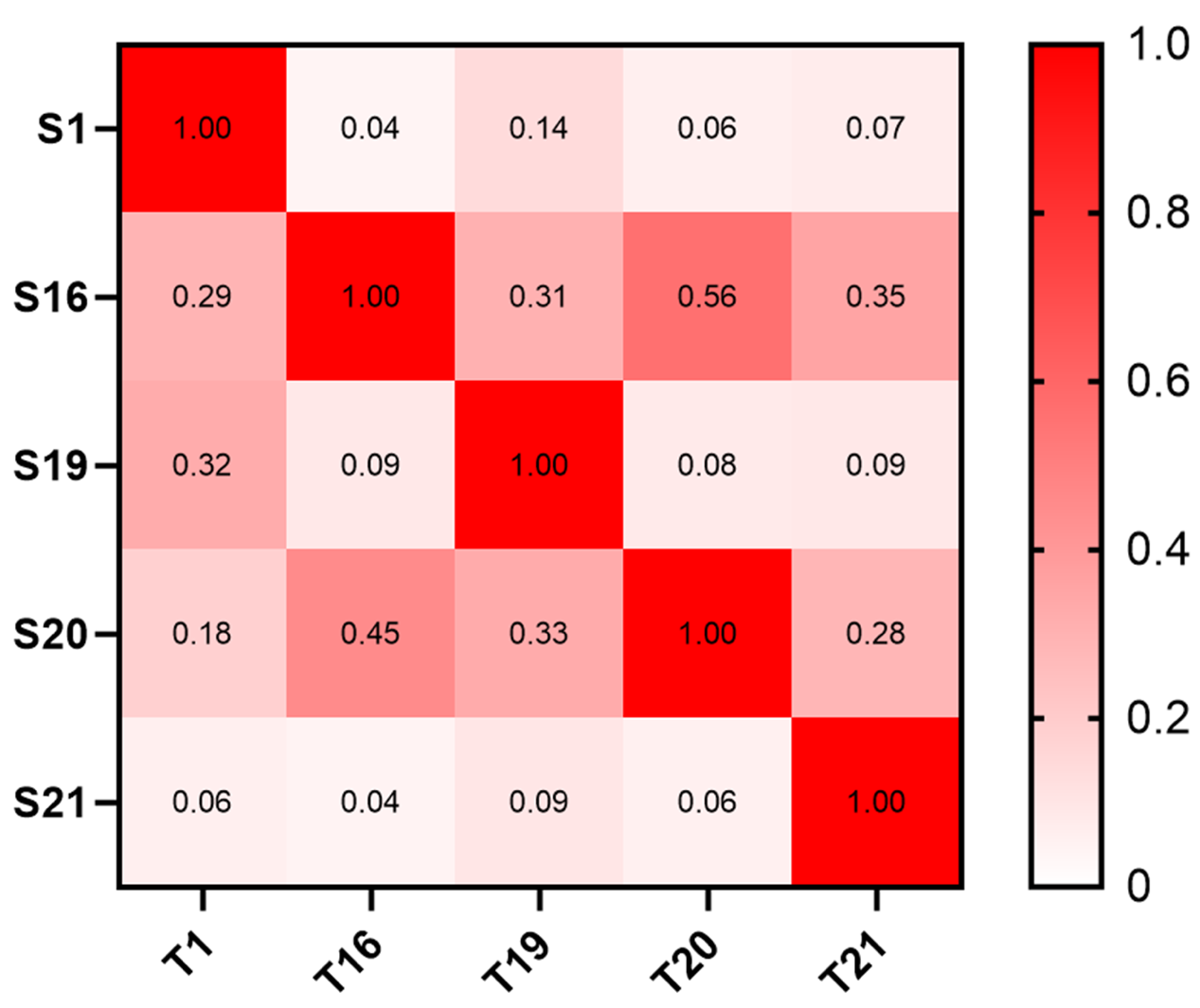


**Supplementary Figure S15. Orthogonality testing of five mutually orthogonal sequences.**

In vitro experimental characterization of the five orthogonal SWTs generated by the optimized algorithm. Each element of the matrix represents the ON/OFF ratio for the indicated SWT/trigger combination. Fold change value was represented by a color scale. Experiment setup: Plasmid-sensor final concentration 1.5 nM; Linear DNA-trigger final concentration 20 nM; Reaction time 2 hours; Temperature 37 °C; Endpoint measurement fluorescence = fluorescence at T = 2h – fluorescence at T = 0h – background fluorescence; Three repeats for each setup.

**Supplemetary Tables**

**Supplementary Table S1. Examples of DNA plasmid sequences.**

AmpR = Ampicilin resistance cassettes.

| Name | Sequence |
| --- | --- |
| Example Sensor Plasmid: Sensor-1(T7 promoter-S1-21linker-3WJDB-T7 terminator-ColE1 origin-AmpR) | TAATACGACTCACTATAGGGAAGCACCACATTCCTCATCACCCCCCTTCCTCACACTTTTCACAAAAGCCCGCCGAAAGGCGGGCTTTTTTTTGCAACCTGGCGGCAGCGCAAAAGAGAGGAGAACTCGGACCCACATACTCTGATGATCCGAGACGGTCGGGTCCAGATATTCGTATCTGTCGAGTAGAGTGTGGGCTCGGATCATTCATGGCAAGAGACGGTCGGGTCCAGATATTCGTATCTGTCGAGTAGAGTGTGGGCTCTTGCCATGTGTATGTGGGGACTCTAGCATAACCCCTTGGGGCCTCTAAACGGGTCTTGAGGGGTTTTTTGGTCGCTCACTCAAAGGCGGTAATACGGTTATCCACAGAATCAGGGGATAACGCAGGAAAGAACATGTGAGCAAAAGGCCAGCAAAAGGCCAGGAACCGTAAAAAGGCCGCGTTGCTGGCGTTTTTCCATAGGCTCCGCCCCCCTGACGAGCATCACAAAAATCGACGCTCAAGTCAGAGGTGGCGAAACCCGACAGGACTATAAAGATACCAGGCGTTTCCCCCTGGAAGCTCCCTCGTGCGCTCTCCTGTTCCGACCCTGCCGCTTACCGGATACCTGTCCGCCTTTCTCCCTTCGGGAAGCGTGGCGCTTTCTCATAGCTCACGCTGTAGGTATCTCAGTTCGGTGTAGGTCGTTCGCTCCAAGCTGGGCTGTGTGCACGAACCCCCCGTTCAGCCCGACCGCTGCGCCTTATCCGGTAACTATCGTCTTGAGTCCAACCCGGTAAGACACGACTTATCGCCACTGGCAGCAGCCACTGGTAACAGGATTAGCAGAGCGAGGTATGTAGGCGGTGCTACAGAGTTCTTGAAGTGGTGGCCTAACTACGGCTACACTAGAAGAACAGTATTTGGTATCTGCGCTCTGCTGAAGCCAGTTACCTTCGGAAAAAGAGTTGGTAGCTCTTGATCCGGCAAACAAACCACCGCTGGTAGCGGTGGTTTTTTTGTTTGCAAGCAGCAGATTACGCGCAGAAAAAAAGGATCTCAAGAAGATCCTTTGATCTTTTCTACGGGGTCTGACGCTCAGTGGAACGAAAACTCACGTTAAGGGATTTTGGTCATGAGATTATCAAAAAGGATCTTCACCTAGATCCTTTTAAATTAAAAATGAAGTTTTAAATCAATCTAAAGTATATATGAGTAAACTTGGTCTGACAGTTACCAATGCTTAATCAGTGAGGCACCTATCTCAGCGATCTGTCTATTTCGTTCATCCATAGTTGCCTGACTCCCCGTCGTGTAGATAACTACGATACGGGAGGGCTTACCATCTGGCCCCAGTGCTGCAATGATACCGCGTGACCCACGCTCACCGGCTCCAGATTTATCAGCAATAAACCAGCCAGCCGGAAGGGCCGAGCGCAGAAGTGGTCCTGCAACTTTATCCGCCTCCATCCAGTCTATTAATTGTTGCCGGGAAGCTAGAGTAAGTAGTTCGCCAGTTAATAGTTTGCGCAACGTTGTTGCCATTGCTACAGGCATCGTGGTGTCACGCTCGTCGTTTGGTATGGCTTCATTCAGCTCCGGTTCCCAACGATCAAGGCGAGTTGCATGATCCCCCATGTTGTGCAAAAAAGCGGTTAGCTCCTTCGGTCCTCCGATCGTTGTCAGAAGTAAGTTGGCCGCAGTGTTATCACTCATGGTTATGGCAGCACTGCATAATTCTCTTACTGTCATGCCATCCGTAAGATGCTTTTCTGTGACTGGTGAGTACTCAACCAAGTCATTCTGAGAATAGTGTATGCGGCGACCGAGTTGCTCTTGCCCGGCGTCAACACGGGATAATACCGCGCCACATAGCAGAACTTTAAAAGTGCTCATCATTGGAAAACGTTCTTCGGGGCGAAAACTCTCAAGGATCTTACCGCTGTTGAGATCCAGTTCGATGTAACCCACTCGTGCACCCAACTGATCTTCAGCATCTTTTACTTTCACCAGCGTTTCTGGGTGAGCAAAAACAGGAAGGCAAAATGCCGCAAAAAAGGGAATAAGGGCGACACGGAAATGTTGAATACTCAT |
| Example Trigger Plasmid: Trigger-1(T7 promoter-T1-T7 terminator-ColE1 origin-AmpR) | TAATACGACTCACTATAGGGAAGCGGCGGGCTTTTGTGAAAAGTGTGAGGAAGGGGGGTGATGAGGAATGTGGTTTGCCTAGCATAACCCCTTGGGGCCTCTAAACGGGTCTTGAGGGGTTTTTTGGTCGCTCACTCAAAGGCGGTAATACGGTTATCCACAGAATCAGGGGATAACGCAGGAAAGAACATGTGAGCAAAAGGCCAGCAAAAGGCCAGGAACCGTAAAAAGGCCGCGTTGCTGGCGTTTTTCCATAGGCTCCGCCCCCCTGACGAGCATCACAAAAATCGACGCTCAAGTCAGAGGTGGCGAAACCCGACAGGACTATAAAGATACCAGGCGTTTCCCCCTGGAAGCTCCCTCGTGCGCTCTCCTGTTCCGACCCTGCCGCTTACCGGATACCTGTCCGCCTTTCTCCCTTCGGGAAGCGTGGCGCTTTCTCATAGCTCACGCTGTAGGTATCTCAGTTCGGTGTAGGTCGTTCGCTCCAAGCTGGGCTGTGTGCACGAACCCCCCGTTCAGCCCGACCGCTGCGCCTTATCCGGTAACTATCGTCTTGAGTCCAACCCGGTAAGACACGACTTATCGCCACTGGCAGCAGCCACTGGTAACAGGATTAGCAGAGCGAGGTATGTAGGCGGTGCTACAGAGTTCTTGAAGTGGTGGCCTAACTACGGCTACACTAGAAGAACAGTATTTGGTATCTGCGCTCTGCTGAAGCCAGTTACCTTCGGAAAAAGAGTTGGTAGCTCTTGATCCGGCAAACAAACCACCGCTGGTAGCGGTGGTTTTTTTGTTTGCAAGCAGCAGATTACGCGCAGAAAAAAAGGATCTCAAGAAGATCCTTTGATCTTTTCTACGGGGTCTGACGCTCAGTGGAACGAAAACTCACGTTAAGGGATTTTGGTCATGAGATTATCAAAAAGGATCTTCACCTAGATCCTTTTAAATTAAAAATGAAGTTTTAAATCAATCTAAAGTATATATGAGTAAACTTGGTCTGACAGTTACCAATGCTTAATCAGTGAGGCACCTATCTCAGCGATCTGTCTATTTCGTTCATCCATAGTTGCCTGACTCCCCGTCGTGTAGATAACTACGATACGGGAGGGCTTACCATCTGGCCCCAGTGCTGCAATGATACCGCGTGACCCACGCTCACCGGCTCCAGATTTATCAGCAATAAACCAGCCAGCCGGAAGGGCCGAGCGCAGAAGTGGTCCTGCAACTTTATCCGCCTCCATCCAGTCTATTAATTGTTGCCGGGAAGCTAGAGTAAGTAGTTCGCCAGTTAATAGTTTGCGCAACGTTGTTGCCATTGCTACAGGCATCGTGGTGTCACGCTCGTCGTTTGGTATGGCTTCATTCAGCTCCGGTTCCCAACGATCAAGGCGAGTTGCATGATCCCCCATGTTGTGCAAAAAAGCGGTTAGCTCCTTCGGTCCTCCGATCGTTGTCAGAAGTAAGTTGGCCGCAGTGTTATCACTCATGGTTATGGCAGCACTGCATAATTCTCTTACTGTCATGCCATCCGTAAGATGCTTTTCTGTGACTGGTGAGTACTCAACCAAGTCATTCTGAGAATAGTGTATGCGGCGACCGAGTTGCTCTTGCCCGGCGTCAACACGGGATAATACCGCGCCACATAGCAGAACTTTAAAAGTGCTCATCATTGGAAAACGTTCTTCGGGGCGAAAACTCTCAAGGATCTTACCGCTGTTGAGATCCAGTTCGATGTAACCCACTCGTGCACCCAACTGATCTTCAGCATCTTTTACTTTCACCAGCGTTTCTGGGTGAGCAAAAACAGGAAGGCAAAATGCCGCAAAAAAGGGAATAAGGGCGACACGGAAATGTTGAATACTCAT |
| S14-T1 in Three-layer cascade Plasmid:(T7 promoter-S14-21linker-T1-T7 terminator-ColE1 origin-AmpR) | TAATACGACTCACTATAGGGAAGCTCCTGAACCCTATATACAACTAATTCTAACACGTCCTCGAAAAGCCCGCCGAAAGGCGGGCTTTTTTTTGCAACCTGGCGGCAGCGCAAAAGGGCGGGCTTTTGTGAAAAGTGTGAGGAAGGGGGGTGATGAGGAATGTGGTTTGCCTAGCATAACCCCTTGGGGCCTCTAAACGGGTCTTGAGGGGTTTTTTGGTCGCTCACTCAAAGGCGGTAATACGGTTATCCACAGAATCAGGGGATAACGCAGGAAAGAACATGTGAGCAAAAGGCCAGCAAAAGGCCAGGAACCGTAAAAAGGCCGCGTTGCTGGCGTTTTTCCATAGGCTCCGCCCCCCTGACGAGCATCACAAAAATCGACGCTCAAGTCAGAGGTGGCGAAACCCGACAGGACTATAAAGATACCAGGCGTTTCCCCCTGGAAGCTCCCTCGTGCGCTCTCCTGTTCCGACCCTGCCGCTTACCGGATACCTGTCCGCCTTTCTCCCTTCGGGAAGCGTGGCGCTTTCTCATAGCTCACGCTGTAGGTATCTCAGTTCGGTGTAGGTCGTTCGCTCCAAGCTGGGCTGTGTGCACGAACCCCCCGTTCAGCCCGACCGCTGCGCCTTATCCGGTAACTATCGTCTTGAGTCCAACCCGGTAAGACACGACTTATCGCCACTGGCAGCAGCCACTGGTAACAGGATTAGCAGAGCGAGGTATGTAGGCGGTGCTACAGAGTTCTTGAAGTGGTGGCCTAACTACGGCTACACTAGAAGAACAGTATTTGGTATCTGCGCTCTGCTGAAGCCAGTTACCTTCGGAAAAAGAGTTGGTAGCTCTTGATCCGGCAAACAAACCACCGCTGGTAGCGGTGGTTTTTTTGTTTGCAAGCAGCAGATTACGCGCAGAAAAAAAGGATCTCAAGAAGATCCTTTGATCTTTTCTACGGGGTCTGACGCTCAGTGGAACGAAAACTCACGTTAAGGGATTTTGGTCATGAGATTATCAAAAAGGATCTTCACCTAGATCCTTTTAAATTAAAAATGAAGTTTTAAATCAATCTAAAGTATATATGAGTAAACTTGGTCTGACAGTTACCAATGCTTAATCAGTGAGGCACCTATCTCAGCGATCTGTCTATTTCGTTCATCCATAGTTGCCTGACTCCCCGTCGTGTAGATAACTACGATACGGGAGGGCTTACCATCTGGCCCCAGTGCTGCAATGATACCGCGTGACCCACGCTCACCGGCTCCAGATTTATCAGCAATAAACCAGCCAGCCGGAAGGGCCGAGCGCAGAAGTGGTCCTGCAACTTTATCCGCCTCCATCCAGTCTATTAATTGTTGCCGGGAAGCTAGAGTAAGTAGTTCGCCAGTTAATAGTTTGCGCAACGTTGTTGCCATTGCTACAGGCATCGTGGTGTCACGCTCGTCGTTTGGTATGGCTTCATTCAGCTCCGGTTCCCAACGATCAAGGCGAGTTGCATGATCCCCCATGTTGTGCAAAAAAGCGGTTAGCTCCTTCGGTCCTCCGATCGTTGTCAGAAGTAAGTTGGCCGCAGTGTTATCACTCATGGTTATGGCAGCACTGCATAATTCTCTTACTGTCATGCCATCCGTAAGATGCTTTTCTGTGACTGGTGAGTACTCAACCAAGTCATTCTGAGAATAGTGTATGCGGCGACCGAGTTGCTCTTGCCCGGCGTCAACACGGGATAATACCGCGCCACATAGCAGAACTTTAAAAGTGCTCATCATTGGAAAACGTTCTTCGGGGCGAAAACTCTCAAGGATCTTACCGCTGTTGAGATCCAGTTCGATGTAACCCACTCGTGCACCCAACTGATCTTCAGCATCTTTTACTTTCACCAGCGTTTCTGGGTGAGCAAAAACAGGAAGGCAAAATGCCGCAAAAAAGGGAATAAGGGCGACACGGAAATGTTGAATACTCAT |
| S19-T17 in Four-layer cascade Plasmid:(T7 promoter-S19-21linker-T17-T7 terminator-ColE1 origin-AmpR) | TAATACGACTCACTATAGGGAAGCCCACGTACTAACTCTCATCACTCACGCACTCATCGACTATAAAGCCCGCCGAAAGGCGGGCTTTTTTTTACAACCTGGCGGCAGCGCAAAAGGGCGGGCTTTATGAACTGGATGGATGGACTGGATGATGGCTTGACGGACGTTACCTAGCATAACCCCTTGGGGCCTCTAAACGGGTCTTGAGGGGTTTTTTGGTCGCTCACTCAAAGGCGGTAATACGGTTATCCACAGAATCAGGGGATAACGCAGGAAAGAACATGTGAGCAAAAGGCCAGCAAAAGGCCAGGAACCGTAAAAAGGCCGCGTTGCTGGCGTTTTTCCATAGGCTCCGCCCCCCTGACGAGCATCACAAAAATCGACGCTCAAGTCAGAGGTGGCGAAACCCGACAGGACTATAAAGATACCAGGCGTTTCCCCCTGGAAGCTCCCTCGTGCGCTCTCCTGTTCCGACCCTGCCGCTTACCGGATACCTGTCCGCCTTTCTCCCTTCGGGAAGCGTGGCGCTTTCTCATAGCTCACGCTGTAGGTATCTCAGTTCGGTGTAGGTCGTTCGCTCCAAGCTGGGCTGTGTGCACGAACCCCCCGTTCAGCCCGACCGCTGCGCCTTATCCGGTAACTATCGTCTTGAGTCCAACCCGGTAAGACACGACTTATCGCCACTGGCAGCAGCCACTGGTAACAGGATTAGCAGAGCGAGGTATGTAGGCGGTGCTACAGAGTTCTTGAAGTGGTGGCCTAACTACGGCTACACTAGAAGAACAGTATTTGGTATCTGCGCTCTGCTGAAGCCAGTTACCTTCGGAAAAAGAGTTGGTAGCTCTTGATCCGGCAAACAAACCACCGCTGGTAGCGGTGGTTTTTTTGTTTGCAAGCAGCAGATTACGCGCAGAAAAAAAGGATCTCAAGAAGATCCTTTGATCTTTTCTACGGGGTCTGACGCTCAGTGGAACGAAAACTCACGTTAAGGGATTTTGGTCATGAGATTATCAAAAAGGATCTTCACCTAGATCCTTTTAAATTAAAAATGAAGTTTTAAATCAATCTAAAGTATATATGAGTAAACTTGGTCTGACAGTTACCAATGCTTAATCAGTGAGGCACCTATCTCAGCGATCTGTCTATTTCGTTCATCCATAGTTGCCTGACTCCCCGTCGTGTAGATAACTACGATACGGGAGGGCTTACCATCTGGCCCCAGTGCTGCAATGATACCGCGTGACCCACGCTCACCGGCTCCAGATTTATCAGCAATAAACCAGCCAGCCGGAAGGGCCGAGCGCAGAAGTGGTCCTGCAACTTTATCCGCCTCCATCCAGTCTATTAATTGTTGCCGGGAAGCTAGAGTAAGTAGTTCGCCAGTTAATAGTTTGCGCAACGTTGTTGCCATTGCTACAGGCATCGTGGTGTCACGCTCGTCGTTTGGTATGGCTTCATTCAGCTCCGGTTCCCAACGATCAAGGCGAGTTGCATGATCCCCCATGTTGTGCAAAAAAGCGGTTAGCTCCTTCGGTCCTCCGATCGTTGTCAGAAGTAAGTTGGCCGCAGTGTTATCACTCATGGTTATGGCAGCACTGCATAATTCTCTTACTGTCATGCCATCCGTAAGATGCTTTTCTGTGACTGGTGAGTACTCAACCAAGTCATTCTGAGAATAGTGTATGCGGCGACCGAGTTGCTCTTGCCCGGCGTCAACACGGGATAATACCGCGCCACATAGCAGAACTTTAAAAGTGCTCATCATTGGAAAACGTTCTTCGGGGCGAAAACTCTCAAGGATCTTACCGCTGTTGAGATCCAGTTCGATGTAACCCACTCGTGCACCCAACTGATCTTCAGCATCTTTTACTTTCACCAGCGTTTCTGGGTGAGCAAAAACAGGAAGGCAAAATGCCGCAAAAAAGGGAATAAGGGCGACACGGAAATGTTGAATACTCAT |
| S17-T18 in Four-layer cascade and two-input three-layer OR gate Plasmid:((T7 promoter-S17-21linker-T18-T7 terminator-ColE1 origin-AmpR)) | TAATACGACTCACTATAGGGAAGCCGTCCGTCAAGCCATCATCCAGTCCATCCATCCAGTTCATAAAGCCCGCCGAAAGGCGGGCTTTTTTTTACAACCTGGCGGCAGCGCAAAAGGGCGGGCTTTGTAAGTTCGTCGTAGTAGCAGCAGGTAGGTCAGGTAGGTCTTACCTAGCATAACCCCTTGGGGCCTCTAAACGGGTCTTGAGGGGTTTTTTGGTCGCTCACTCAAAGGCGGTAATACGGTTATCCACAGAATCAGGGGATAACGCAGGAAAGAACATGTGAGCAAAAGGCCAGCAAAAGGCCAGGAACCGTAAAAAGGCCGCGTTGCTGGCGTTTTTCCATAGGCTCCGCCCCCCTGACGAGCATCACAAAAATCGACGCTCAAGTCAGAGGTGGCGAAACCCGACAGGACTATAAAGATACCAGGCGTTTCCCCCTGGAAGCTCCCTCGTGCGCTCTCCTGTTCCGACCCTGCCGCTTACCGGATACCTGTCCGCCTTTCTCCCTTCGGGAAGCGTGGCGCTTTCTCATAGCTCACGCTGTAGGTATCTCAGTTCGGTGTAGGTCGTTCGCTCCAAGCTGGGCTGTGTGCACGAACCCCCCGTTCAGCCCGACCGCTGCGCCTTATCCGGTAACTATCGTCTTGAGTCCAACCCGGTAAGACACGACTTATCGCCACTGGCAGCAGCCACTGGTAACAGGATTAGCAGAGCGAGGTATGTAGGCGGTGCTACAGAGTTCTTGAAGTGGTGGCCTAACTACGGCTACACTAGAAGAACAGTATTTGGTATCTGCGCTCTGCTGAAGCCAGTTACCTTCGGAAAAAGAGTTGGTAGCTCTTGATCCGGCAAACAAACCACCGCTGGTAGCGGTGGTTTTTTTGTTTGCAAGCAGCAGATTACGCGCAGAAAAAAAGGATCTCAAGAAGATCCTTTGATCTTTTCTACGGGGTCTGACGCTCAGTGGAACGAAAACTCACGTTAAGGGATTTTGGTCATGAGATTATCAAAAAGGATCTTCACCTAGATCCTTTTAAATTAAAAATGAAGTTTTAAATCAATCTAAAGTATATATGAGTAAACTTGGTCTGACAGTTACCAATGCTTAATCAGTGAGGCACCTATCTCAGCGATCTGTCTATTTCGTTCATCCATAGTTGCCTGACTCCCCGTCGTGTAGATAACTACGATACGGGAGGGCTTACCATCTGGCCCCAGTGCTGCAATGATACCGCGTGACCCACGCTCACCGGCTCCAGATTTATCAGCAATAAACCAGCCAGCCGGAAGGGCCGAGCGCAGAAGTGGTCCTGCAACTTTATCCGCCTCCATCCAGTCTATTAATTGTTGCCGGGAAGCTAGAGTAAGTAGTTCGCCAGTTAATAGTTTGCGCAACGTTGTTGCCATTGCTACAGGCATCGTGGTGTCACGCTCGTCGTTTGGTATGGCTTCATTCAGCTCCGGTTCCCAACGATCAAGGCGAGTTGCATGATCCCCCATGTTGTGCAAAAAAGCGGTTAGCTCCTTCGGTCCTCCGATCGTTGTCAGAAGTAAGTTGGCCGCAGTGTTATCACTCATGGTTATGGCAGCACTGCATAATTCTCTTACTGTCATGCCATCCGTAAGATGCTTTTCTGTGACTGGTGAGTACTCAACCAAGTCATTCTGAGAATAGTGTATGCGGCGACCGAGTTGCTCTTGCCCGGCGTCAACACGGGATAATACCGCGCCACATAGCAGAACTTTAAAAGTGCTCATCATTGGAAAACGTTCTTCGGGGCGAAAACTCTCAAGGATCTTACCGCTGTTGAGATCCAGTTCGATGTAACCCACTCGTGCACCCAACTGATCTTCAGCATCTTTTACTTTCACCAGCGTTTCTGGGTGAGCAAAAACAGGAAGGCAAAATGCCGCAAAAAAGGGAATAAGGGCGACACGGAAATGTTGAATACTCAT |
| S19-T18 in Four-layer cascade Plasmid:(T7 promoter-S19-21linker-T18-T7 terminator-ColE1 origin-AmpR) | TAATACGACTCACTATAGGGAAGCCCACGTACTAACTCTCATCACTCACGCACTCATCGACTATAAAGCCCGCCGAAAGGCGGGCTTTTTTTTACAACCTGGCGGCAGCGCAAAAGGGCGGGCTTTGTAAGTTCGTCGTAGTAGCAGCAGGTAGGTCAGGTAGGTCTTACCTAGCATAACCCCTTGGGGCCTCTAAACGGGTCTTGAGGGGTTTTTTGGTCGCTCACTCAAAGGCGGTAATACGGTTATCCACAGAATCAGGGGATAACGCAGGAAAGAACATGTGAGCAAAAGGCCAGCAAAAGGCCAGGAACCGTAAAAAGGCCGCGTTGCTGGCGTTTTTCCATAGGCTCCGCCCCCCTGACGAGCATCACAAAAATCGACGCTCAAGTCAGAGGTGGCGAAACCCGACAGGACTATAAAGATACCAGGCGTTTCCCCCTGGAAGCTCCCTCGTGCGCTCTCCTGTTCCGACCCTGCCGCTTACCGGATACCTGTCCGCCTTTCTCCCTTCGGGAAGCGTGGCGCTTTCTCATAGCTCACGCTGTAGGTATCTCAGTTCGGTGTAGGTCGTTCGCTCCAAGCTGGGCTGTGTGCACGAACCCCCCGTTCAGCCCGACCGCTGCGCCTTATCCGGTAACTATCGTCTTGAGTCCAACCCGGTAAGACACGACTTATCGCCACTGGCAGCAGCCACTGGTAACAGGATTAGCAGAGCGAGGTATGTAGGCGGTGCTACAGAGTTCTTGAAGTGGTGGCCTAACTACGGCTACACTAGAAGAACAGTATTTGGTATCTGCGCTCTGCTGAAGCCAGTTACCTTCGGAAAAAGAGTTGGTAGCTCTTGATCCGGCAAACAAACCACCGCTGGTAGCGGTGGTTTTTTTGTTTGCAAGCAGCAGATTACGCGCAGAAAAAAAGGATCTCAAGAAGATCCTTTGATCTTTTCTACGGGGTCTGACGCTCAGTGGAACGAAAACTCACGTTAAGGGATTTTGGTCATGAGATTATCAAAAAGGATCTTCACCTAGATCCTTTTAAATTAAAAATGAAGTTTTAAATCAATCTAAAGTATATATGAGTAAACTTGGTCTGACAGTTACCAATGCTTAATCAGTGAGGCACCTATCTCAGCGATCTGTCTATTTCGTTCATCCATAGTTGCCTGACTCCCCGTCGTGTAGATAACTACGATACGGGAGGGCTTACCATCTGGCCCCAGTGCTGCAATGATACCGCGTGACCCACGCTCACCGGCTCCAGATTTATCAGCAATAAACCAGCCAGCCGGAAGGGCCGAGCGCAGAAGTGGTCCTGCAACTTTATCCGCCTCCATCCAGTCTATTAATTGTTGCCGGGAAGCTAGAGTAAGTAGTTCGCCAGTTAATAGTTTGCGCAACGTTGTTGCCATTGCTACAGGCATCGTGGTGTCACGCTCGTCGTTTGGTATGGCTTCATTCAGCTCCGGTTCCCAACGATCAAGGCGAGTTGCATGATCCCCCATGTTGTGCAAAAAAGCGGTTAGCTCCTTCGGTCCTCCGATCGTTGTCAGAAGTAAGTTGGCCGCAGTGTTATCACTCATGGTTATGGCAGCACTGCATAATTCTCTTACTGTCATGCCATCCGTAAGATGCTTTTCTGTGACTGGTGAGTACTCAACCAAGTCATTCTGAGAATAGTGTATGCGGCGACCGAGTTGCTCTTGCCCGGCGTCAACACGGGATAATACCGCGCCACATAGCAGAACTTTAAAAGTGCTCATCATTGGAAAACGTTCTTCGGGGCGAAAACTCTCAAGGATCTTACCGCTGTTGAGATCCAGTTCGATGTAACCCACTCGTGCACCCAACTGATCTTCAGCATCTTTTACTTTCACCAGCGTTTCTGGGTGAGCAAAAACAGGAAGGCAAAATGCCGCAAAAAAGGGAATAAGGGCGACACGGAAATGTTGAATACTCAT |

**Supplementary Table S2. Sensor sequences used in this study.**

Plasmid sequences can be constructed by replacing the purple region in the example sensor plasmid in **Supplementary Table 1** with the purple region indicated here.

| Sensor sequence | Name | Figure |
| --- | --- | --- |
| ACCACATTCCTCATCACCCCCCTTCCTCACACTTTTCACAAAAGCCCGCCGAAAGGCGGGCTTTTTTTT | S1 (50%) | 2,3,S2,S4,S6,S9 |
| ATCTCTTCCTCTCTCCTCACCACCCCAACCATCCCAGCCCAAAGCCCGCCGAAAGGCGGGCTTTTTTTT | S2 (60%) | 3,S2 |
| TCCCTCCCCCCAACTCATCCTCAACCTCAAACTCACACACAAAGCCCGCCGAAAGGCGGGCTTTTTTTT | S3 (55%) | 3,S2 |
| GACAACAACCCAACACACACAAAACCACACAAAACCACAGAAAGCCCGCCGAAAGGCGGGCTTTTTTTT | S4 (45%) | 3,S2 |
| ATCTCATAACTACCATCTCCATACCTCATTCCTCTTCCTAAAAGCCCGCCGAAAGGCGGGCTTTTTTTT | S5 (40%) | 3,S2 |
| TCAACACAATCAATCAACACCTCTACAAAACACTACACAAAAAGCCCGCCGAAAGGCGGGCTTTTTTTT | S6 (35%) | 3,4,S2 |
| TCAACACAATCAATCAACACCTCTACAAAACACTACACAAAAAGCGCGGCCGGGAAACCGGCCGCGCTTTTTTTT | S7 (10 nt) | 4 |
| TCAACACAATCAATCAACACCTCTACAAAACACTACACAAAAAGGCGGCCGCGGCCGAAAGGCCGCGGCCGCCTTTTTTTT | S8 (13 nt) | 4 |
| TCAACACAATCAATCAACACCTCTACAAAACACTACACAAAAACCGGCCGGCGGGCGCGGAAACGCGCCCGCCGGCCGGTTTTTTTT | S9 (16 nt) | 4 |
| TCAACACAATCAATCAACACCTCTACAAAACACTACACAAAAACGGCCCCTCGGTAACGAGGGGCCGTTTTTTTT | S10 (90%) | 4 |
| TCAACACAATCAATCAACACCTCTACAAAACACTACACAAAAAGCAGGCGTGCGTAAGCACGCCTGCTTTTTTTT | S11 (80%) | 4 |
| TCAACACAATCAATCAACACCTCTACAAAACACTACACAAAAACGGGTGCACTGTAAAGTGCACCCGTTTTTTTT | S12 (70%) | 4 |
| ACCACATTCCTCATCACCCCCCTTCCTCACACTTTTCACAAAACGGCCCCTCGGTAACGAGGGGCCGTTTTTTTT | S13 | 4,S3 |
| TCCTGAACCCTATATACAACTAATTCTAACACGTCCTCGAAAAGCCCGCCGAAAGGCGGGCTTTTTTTT | S14 | S4,S5,S6 |
| GTCATCCGTCATCCATTCGTCATCCATCGTTCGCTCATCCAAAGCCCGCCGAAAGGCGGGCTTTTTTTT | S15 | 5,S7 |
| GACCTACCTACTACTACTACCTACTGACCTACGTGCCTACAAAGCCCGCCGAAAGGCGGGCTTTTTTTT | S16 | 5,S7,S9 |
| CGTCCGTCAAGCCATCATCCAGTCCATCCATCCAGTTCATAAAGCCCGCCGAAAGGCGGGCTTTTTTTT | S17 | 6, 7, S8 |
| GACCTACCTGACCTACCTGCTGCTACTACGACGAACTTACAAAGCCCGCCGAAAGGCGGGCTTTTTTTT | S18 | 6, 7, S8 |
| CCACGTACTAACTCTCATCACTCACGCACTCATCGACTATAAAGCCCGCCGAAAGGCGGGCTTTTTTTT | S19 | 6, 7, S8,S9 |
| GACCTACCTACTACTACTACCTACTGACCTACGTGCCTACAAAGCCCGCCGAAAGGCGGGCTTTTTTTT | S20 | S9 |
| CTGACCCGAGCAACCACCTATACCTTAAACCGACACTAGCAAAGCCCGCCGAAAGGCGGGCTTTTTTTT | S21 | S9 |

**Supplementary Table S3. Trigger sequences used in this study.**

Plasmid sequences can be constructed by replacing the purple region in the example trigger plasmid in **Supplementary Table 1** with the purple region indicated here.

| Trigger sequence | Name | Figure |
| --- | --- | --- |
| TGTGAAAAGTGTGAGGAAGGGGGGTGATGAGGAATGTGGT | Trigger-(1) | 2 |
| GGCGGGCTTT | Trigger-(2) | 2 |
| GGCGGGCTTTTGTGAAAAGTGTGAGGAAGGGGGGTGATGAGGAATGTGGT | Trigger-(3) | 2 |
| TTTCGGCGGGCTTTTGTGAAAAGTGTGAGGAAGGGGGGTGATGAGGAATGTGGT | Trigger-(4) | 2 |
| GGCGGGCTTTTGTGAAAAGTGTGAGGAAGGGGGGTGATGAGGAATGTGGT | T1 | 3,S2,S4,S5,S6,S9 |
| GGCGGGCTTTGGGCTGGGATGGTTGGGGTGGTGAGGAGAGAGGAAGAGAT | T2 | 3,S2 |
| GGCGGGCTTTGTGTGTGAGTTTGAGGTTGAGGATGAGTTGGGGGGAGGGA | T3 | 3,S2 |
| GGCGGGCTTTCTGTGGTTTTGTGTGGTTTTGTGTGTGTTGGGTTGTTGTC | T4 | 3,S2 |
| GGCGGGCTTTTAGGAAGAGGAATGAGGTATGGAGATGGTAGTTATGAGAT | T5 | 3,S2 |
| GGCGGGCTTTTTGTGTAGTGTTTTGTAGAGGTGTTGATTGATTGTGTTGA | T6 | 3,4,S2 |
| CCGGCCGCGCTTTTTGTGTAGTGTTTTGTAGAGGTGTTGATTGATTGTGTTGA | T7 | 4 |
| GGCCGCGGCCGCCTTTTTGTGTAGTGTTTTGTAGAGGTGTTGATTGATTGTGTTGA | T8 | 4 |
| CGCGCCCGCCGGCCGGTTTTTGTGTAGTGTTTTGTAGAGGTGTTGATTGATTGTGTTGA | T9 | 4 |
| CGAGGGGCCGTTTTTGTGTAGTGTTTTGTAGAGGTGTTGATTGATTGTGTTGA | T10 | 4 |
| GCACGCCTGCTTTTTGTGTAGTGTTTTGTAGAGGTGTTGATTGATTGTGTTGA | T11 | 4 |
| AGTGCACCCGTTTTTGTGTAGTGTTTTGTAGAGGTGTTGATTGATTGTGTTGA | T12 | 4 |
| CGAGGGGCCGTTTTGTGAAAAGTGTGAGGAAGGGGGGTGATGAGGAATGTGGT | T13 | 4,S3 |
| GGCGGGCTTTTCGAGGACGTGTTAGAATTAGTTGTATATAGGGTTCAGGA | T14 | S4,S6 |
| GGCGGGCTTTGGATGAGCGAACGATGGATGACGAATGGATGACGGATGAC | T15 | 5,S7 |
| GGCGGGCTTTGTAGGCACGTAGGTCAGTAGGTAGTAGTAGTAGGTAGGTC | T16 | 5,S7,S9 |
| GGCGGGCTTTATGAACTGGATGGATGGACTGGATGATGGCTTGACGGACG | T17 | 6, 7, S8 |
| GGCGGGCTTTGTAAGTTCGTCGTAGTAGCAGCAGGTAGGTCAGGTAGGTC | T18 | 6, 7, S8 |
| GGCGGGCTTTATAGTCGATGAGTGCGTGAGTGATGAGAGTTAGTACGTGG | T19 | 6, 7, S8,S9 |
| GGCGGGCTTTGTAGGCACGTAGGTCAGTAGGTAGTAGTAGTAGGTAGGTC | T20 | S9 |
| GGCGGGCTTTGCTAGTGTCGGTTTAAGGTATAGGTGGTTGCTCGGGTCAG | T21 | S9 |

**REFERENCES**

1. Zadeh JN, Wolfe BR, Pierce NA. Nucleic acid sequence design via efficient ensemble defect optimization. Journal of computational chemistry*.* 2011;32(3):439-52.

2. Wolfe BR, Porubsky NJ, Zadeh JN, Dirks RM, Pierce NA. Constrained multistate sequence design for nucleic acid reaction pathway engineering. Journal of the American Chemical Society*.* 2017;139(8):3134-44.

3. Wolfe BR, Pierce NA. Sequence design for a test tube of interacting nucleic acid strands. ACS Synthetic Biology*.* 2015;4(10):1086-100.

4. Zadeh JN, Steenberg CD, Bois JS, Wolfe BR, Pierce MB, Khan AR, Dirks RM, Pierce NA. NUPACK: Analysis and design of nucleic acid systems. Journal of computational chemistry*.* 2011;32(1):170-73.
